# A genome–wide CRISPR activation screen identifies *SCREEM* a novel *SNAI1* super-enhancer demarcated by eRNAs

**DOI:** 10.3389/fmolb.2023.1110445

**Published:** 2023-02-27

**Authors:** Dinesh Babu Uthaya Kumar, Marina Yurieva, Jessica Grassmann, Lina Kozhaya, Caleb Dante McBride, Derya Unutmaz, Adam Williams

**Affiliations:** ^1^ The Jackson Laboratory for Genomic Medicine, Farmington, CT, United States; ^2^ The Department of Genetics and Genome Sciences, University of Connecticut Health Center, Farmington, CT, United States; ^3^ Department of Medicine, Division of Allergy and Immunology, Northwestern University Feinberg School of Medicine, Chicago, IL, United States

**Keywords:** CRISPR-screen, EMT, lncRNA, eRNA, SNAI1, CD44, monocytes

## Abstract

The genome is pervasively transcribed to produce a vast array of non-coding RNAs (ncRNAs). Long non-coding RNAs (lncRNAs) are transcripts of >200 nucleotides and are best known for their ability to regulate gene expression. Enhancer RNAs (eRNAs) are subclass of lncRNAs that are synthesized from enhancer regions and have also been shown to coordinate gene expression. The biological function and significance of most lncRNAs and eRNAs remain to be determined. Epithelial to mesenchymal transition (EMT) is a ubiquitous cellular process that occurs during cellular migration, homeostasis, fibrosis, and cancer-cell metastasis. EMT-transcription factors, such as SNAI1 induce a complex transcriptional program that coordinates the morphological and molecular changes associated with EMT. Such complex transcriptional programs are often subject to coordination by networks of ncRNAs and thus can be leveraged to identify novel functional ncRNA loci. Here, using a genome-wide CRISPR activation (CRISPRa) screen targeting ∼10,000 lncRNA loci we identified ncRNA loci that could either promote or attenuate EMT. We discovered a novel locus that we named *SCREEM* (*SNAI1* cis-regulatory eRNAs expressed in monocytes). The *SCREEM* locus contained a cluster of eRNAs that when activated using CRISPRa induced expression of the neighboring gene *SNAI1*, driving concomitant EMT. However, the *SCREEM* eRNA transcripts themselves appeared dispensable for the induction of *SNAI1* expression. Interestingly, the *SCREEM* eRNAs and *SNAI1* were co-expressed in activated monocytes, where the *SCREEM* locus demarcated a monocyte-specific super-enhancer. These findings suggest a potential role for SNAI1 in monocytes. Exploration of the *SCREEM-SNAI* axis could reveal novel aspects of monocyte biology.

## Introduction

The human genome is pervasively transcribed producing a vast array of non-coding RNAs (ncRNAs), most with unknown function (Pertea, 2012; Hangauer et al., 2013; Palazzo and Lee, 2015). Based on size, ncRNAs are broadly classified into small non-coding RNAs (≤200 nucleotides) and long non-coding RNAs (lncRNAs; >200 nucleotides) (Uthaya Kumar and Williams, 2020). Importantly this definition is arbitrary and different ncRNA classes may span this size cutoff. Several evolutionarily conserved classes of ncRNAs, such as miRNAs, rRNAs and tRNAs, have discrete well described functions (Carthew and Sontheimer, 2009; Iwasaki et al., 2015; Jorjani et al., 2016; Treiber et al., 2019). In contrast, many lncRNAs have little to no sequence conservation, and for most, their function and biological relevance remain enigmatic (Rinn and Chang, 2012). Nonetheless, the limited number of lncRNAs that have been well characterized display diverse molecular functions, including but not limited to, the regulation of transcription and translation, coordination of cell signaling, and modulation of metabolic enzymes (Wilusz et al., 2009; Ma et al., 2013). Enhancer RNAs (eRNAs) are a subclass of lncRNAs that were originally characterized as non-polyadenylated and unspliced, bidirectionally transcribed transcripts of under 2kb, that were synthesized from H3K4me1 marked active enhancers (De Santa et al., 2010; Kim et al., 2010). However, the classification of eRNAs has evolved to include enhancer derived RNAs that may be polyadenylated, spliced, unidirectionally transcribed, and over 4 kb in length (Koch et al., 2011). Although the function of most eRNAs is unknown, they have been shown to regulate transcription, facilitate enhancer–promoter interactions, and to modify chromatin accessibility (Li et al., 2016a; Arnold et al., 2019; Lewis et al., 2019; Hou and Kraus, 2021). Regardless of function, eRNAs mark the genomic location of enhancers, and their expression has been used as a proxy to identify enhancers that are currently active (Sartorelli and Lauberth, 2020). Super-enhancers were recently identified as unusually large enhancers with potent cell-type-specific activity (Hnisz et al., 2013). Many super-enhancers are thought to control expression of genes important in specifying and maintaining cell identity (Whyte et al., 2013). Interestingly, super-enhancers are enriched with eRNAs, and these super-enhancer associated eRNAs may play a functional role in super-enhancer biology (Chen and Liang, 2020).

Current methods for predicting the function of lncRNAs are limited; therefore, determining their function is dependent on direct experimental assays. Genome-wide CRISPR screens provide a systematic and scalable approach for interrogation of functional ncRNA loci. However, lncRNAs are frequently more tissue specific than protein-coding genes and so only a subset is expressed in any given cell type; CRISPR activation (CRISPRa) screens overcome this limitation.

Epithelial-mesenchymal transition (EMT) is a cellular process during which epithelial cells trans-differentiate to acquire mesenchymal phenotypes and characteristics following downregulation of epithelial features (Yang et al., 2020). EMT is triggered either by stimulus from the microenvironment or epithelial-cell intrinsic mutations. EMT-transcription factors (TFs) are capable of inducing EMT in normal epithelial cells. Master regulator EMT-TFs such as SNAI1, SNAI2, TWIST1, MZF1, ZEB1 and ZEB2, cooperate with one another to induce a complex transcriptional program that coordinates the morphological and molecular changes associated with EMT (Stemmler et al., 2019). Such complex transcriptional programs are often subject to regulation by ncRNAs. Indeed, several lncRNAs have already been identified to coordinate EMT in various cell types and disease states (Beltran et al., 2008; Orom et al., 2010; Yuan et al., 2014; Li et al., 2016b; Jia et al., 2016; Jin et al., 2016; Liu and Lin, 2016; Grelet et al., 2017; Li et al., 2017; Wu et al., 2017). We therefore leveraged the EMT program to enable identification of novel functional ncRNA loci. In primary bronchial epithelial cells, we performed a genome-wide screen using a Cas9 synergistic activation mediator (SAM) based CRISPR-activation (CRISPRa) approach using a sgRNA library targeting 10,504 intergenic lncRNA loci (Joung et al., 2017a). By exploiting cell surface levels of CD44 as a marker to differentiate between the epithelial-like and mesenchymal-like states, we were able to screen for candidate lncRNAs which promoted or restrained EMT. Among the EMT regulating lncRNAs, we discovered a novel locus that we named *SCREEM* (*SNAI1 cis*-regulatory eRNAs expressed in monocytes). The *SCREEM* locus contained a cluster of eRNAs that were co-expressed with the neighboring EMT-TF *SNAI1*. Targeting *SCREEM* locus with the SAM system activated eRNA expression and induced expression of *SNAI1*, resulting global transcriptional reprogramming and EMT. However, the *SCREEM* eRNA transcripts themselves appeared dispensable for the induction of *SNAI1* expression. Unexpectedly we found that the *SCREEM-SNAI* axis was active in monocytes, where the *SCREEM* locus marked the location of a monocyte super-enhancer. Although SNAI1 is known to be expressed by monocytes, its function in this cell type is currently unknown. Exploration of the *SCREEM-SNAI* axis could reveal novel aspects of monocyte biology. Indeed, other chief EMT-TFs have recently been shown to have important immune modulating functions outside of their traditional roles in EMT (Niesner et al., 2008; Pham et al., 2012; Scott and Omilusik, 2019). In summary, this study demonstrates the value of genome-wide CRISPRa screens in identifying functional ncRNA loci with unique and unexpected biology.

## Results

### A CRISPRa screen identifies candidate lncRNAs in EMT regulation

**FIGURE 1:**
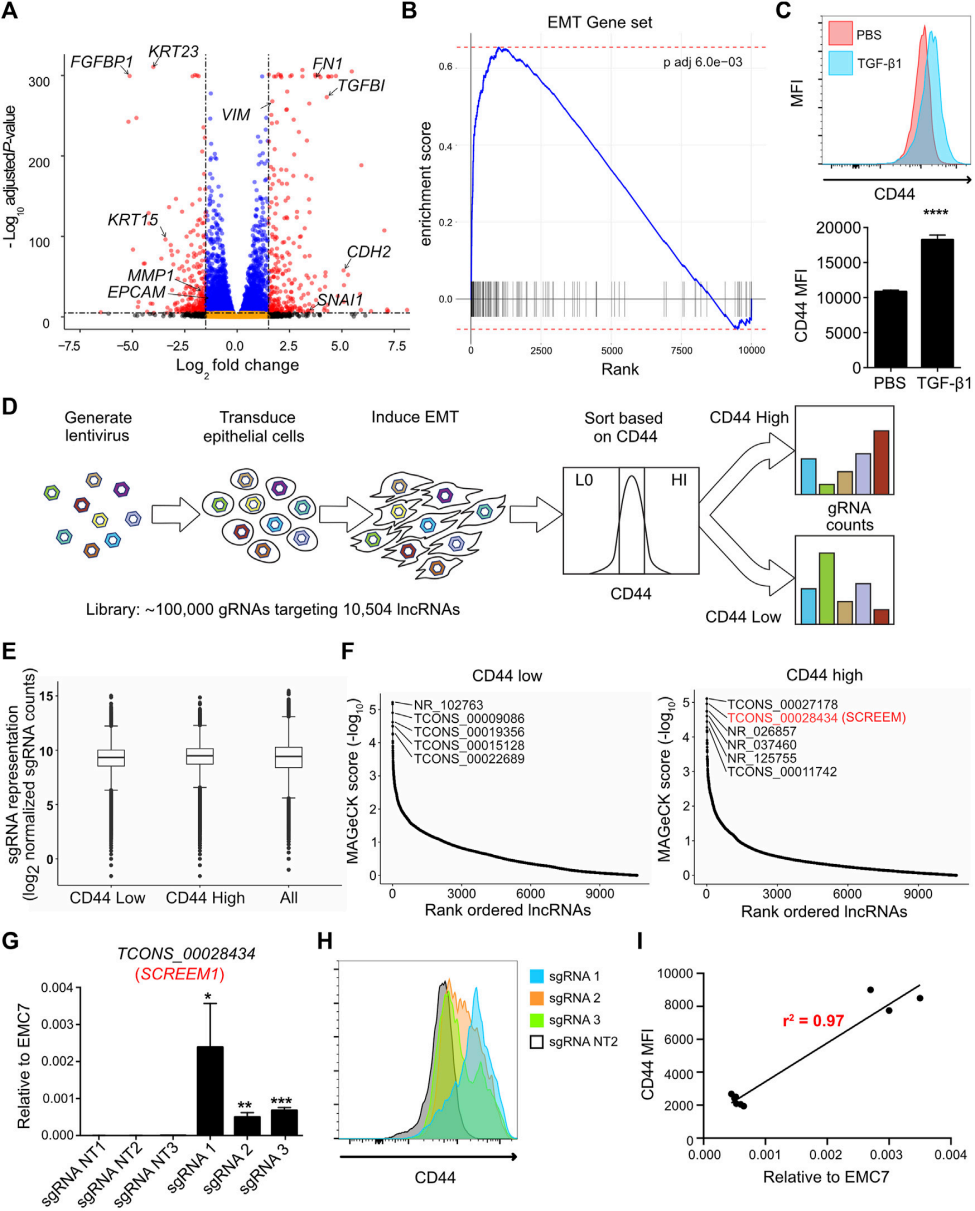
CRISPRa screen identifies functional candidate lncRNA loci. **(A)** Volcano plot of expressed genes (TPM≥1) between PBS and TGFB1 (10ng/ml) treated HBEC3-KT cells, n = 3. Red dots, adjusted *p*-value < 10^–6^ and log2 fold change >2; blue dots, adjusted *p*-value < 10^–6^ and log2 fold change <2; black dots, adjusted *p*-value > 10^–6^ and log2 fold change >2; orange dots, >10^–6^ and log2 fold change <2. **(B)** Ranked ordered gene set enrichment analysis of differentially expressed genes between PBS and TGFB1 (10ng/ul) treated HBEC3-KT cells, n = 3. The EMT pathway is shown. **(C)** Top panel - flow cytometric analysis of cell surface marker expression using CD44 staining, comparing HBEC3-KT cells treated with TGFβ1 (10 ng/ul) or PBS for 72 hrs. Bottom panel - graphs representing flow cytometric results for each replicate. Mean fluorescence intensity (MFI) of CD44 staining is shown. Error Bars, mean ± SD; n = 3. Stats, unpaired *t*-test; ****p < 0.0001 **(D)** Schematic of CRISPRa screen strategy. Cas9 synergistic activation mediator (SAM) based CRISPR activation (CRISPRa) screen; where, 10,504 intergenic lncRNA loci were targeted using a single guide RNA (sgRNA) library (with ∼10 sgRNAs per TSS). Cell surface receptor CD44 was used as a marker to differentiate between epithelial-like (CD44-low) and mesenchymal-like states (CD44-high) after EMT induction using TGFβ1. FACS was used to sort the top 10% high and 10% low the cells based on CD44 expression. **(E)** Box and whisker plot showing sgRNA frequencies before flow sort (all) and after flow sort (CD44 low and CD44 high). Plotted mean counts from n = 3. **(F)** Ranked ordered dot plot of MAGeCK *p* values(−log_10_) for the CD44 low (left) and CD44 high (right). **(G)** RT-PCR analysis *SCREEM1* expression relative to *EMC7* in HBEC3-KT cells upon CRISPR-activation of the *SCREEM1* locus with three different sgRNAs. NT, non-targeting. All values are mean ± SD with n = 3 per sgRNA; unpaired *t*-test with Welch’s correction; *p < 0.05; **p < 0.01; ***p < 0.001. **(H)** Flow cytometric analysis of CD44 expression in HBEC3-KT cells upon CRISPR-activation of the *SCREEM1* locus with different sgRNAs. **(I)** Correlation between relative levels of *SCREEM1* (RT-PCR, panel **(G)** and surface levels of CD44 (flow cytometry, panel H) following activation of the *SCREEM1* locus. Statistics, simple linear regression.

We first sought to establish a genome-wide screen to identify functional lncRNA loci. Since EMT is a complex biological phenomenon that requires changes at transcriptional, translational, and post-translational levels, we asked, whether functional lncRNA loci could be identified by exploiting EMT as a readout. Towards this end, we first solicited a primary epithelial cell line suitable for such a screen. HBEC3-KT cells are normal human primary bronchial epithelial cells immortalized with CDK4 and hTERT (Ramirez et al., 2004). What makes the HBEC3-KT line an attractive epithelial cell line is its potential to trans-differentiate into multiple epithelial subtypes (Vaughan et al., 2006), its lack of mutational burden (Ramirez et al., 2004), and finally its ability to effectively undergo EMT (Figures 1A, B; and Supplementary Table S1). Next, we pursued a cell surface marker that would allow discrimination of epithelial-like and mesenchymal-like cells *via* cell sorting. We generated a comprehensive list of epithelial cell surface markers by cross referencing the cell surface protein atlas (Bausch-Fluck et al., 2015) and our transcriptome data from primary epithelial cells following EMT induction (Uthaya Kumar et al., 2022). We then prioritized cell surface proteins that were highly expressed, detectable by flow-cytometry, and differentially expressed between epithelial and mesenchymal states. Through this strategy, we identified that the cell surface adhesion receptor CD44 was consistently upregulated on HBEC3-KT cells following EMT (Figure 1C). CD44 is a transmembrane glycoprotein with multiple biological functions; however, its primary role is to bind various ligands on extracellular matrix to mediate cellular migration and invasion processes (Ponta et al., 2003; Thorne et al., 2004). For lncRNA targeting we selected the Cas9 synergistic activation mediator (SAM)-based CRISPR-activation (CRISPRa) system and a single guide RNA (sgRNA) library targeting 10,504 intergenic lncRNA loci with approximately 10 sgRNAs per transcriptional start site (TSS) (Joung et al., 2017a). The full sgRNA lentiviral library (Supplementary Figures S1A, B; Supplementary Table S2) was transduced into the HBEC3-KT cells that were engineered to express dCAS9-VP64 and P65 (Konermann et al., 2015), and cultured under antibiotic selection for 14 days. CRISPRa cells were treated with TGFβ1 (10 ng/ml) for 72 h and subsequently flow sorted based on CD44 expression. We took the CD44 low population to represent more epithelial-like cells and the CD44 high population to represent more mesenchymal-like cells (Supplementary Figure S1C), and then assayed for sgRNA enrichment by sequencing (Figure 1D). Distribution of overall sgRNA frequency remained stable over the course of the screen (Figure 1E and Supplementary Table S3), indicating that CRISPRa targeting of lncRNA loci did not exhibit broad non-specific toxicity. Model-based Analysis of Genome-wide CRISPR/Cas9 Knockout (MAGeCK) (Li et al., 2014) identified candidate loci that were significantly enriched (FDR <0.05) in either CD44 low or CD44 high cells (Figure 1F and Supplementary Table S4). To validate the screening results, we individually expressed the three most enriched sgRNAs, targeting candidate lncRNA loci in CD44 low and CD44 high cells (Supplementary Figure S1D). In all 6 cases, the sgRNAs conferred significant lncRNA activation (*p* < 0.01) (Supplementary Figure S1E). Non-etheless, upregulation of candidate lncRNAs enriched in CD44-low population did not result in substantial CD44 reduction either with (data not shown) or without TGFβ1 treatment (Supplementary Figure S1F). In contrast, upregulation of candidate lncRNAs enriched in CD44-high population resulted in significant CD44 induction with (data not shown) or without TGFβ1 treatment (Supplementary Figure S1F). Activation of the *TCONS_0002834* (NONHSAG031990) locus (here after referred to as the *SCREEM1* locus) by three different sgRNAs (Figure 1G), consistently demonstrated upregulation of CD44 either in the presence (data not shown) or absence of TGFβ1 (Figure 1H). Furthermore, the relative expression of *SCREEM1* in HBEC3-KT cells correlated with the concomitant increase in CD44 expression (*r* ^2^ = 0.97) (Figure 1I).

### Activation of the *SCREEM1* locus elicits a robust transcriptional reprograming

**FIGURE 2:**
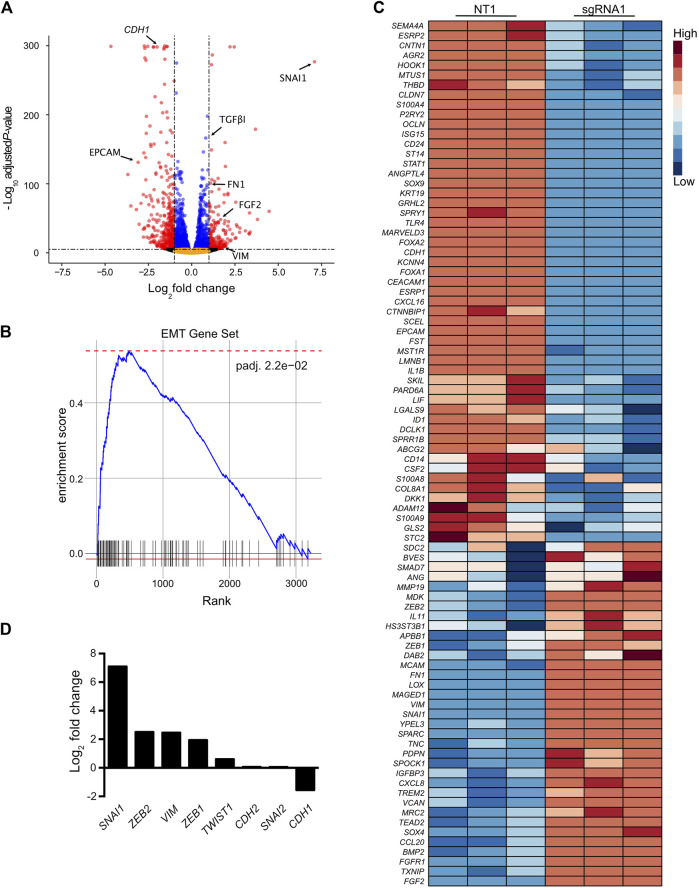
Activation of the *SCREEM1* locus elicits a robust transcriptional reprogramming. **(A)** Volcano plot of expressed genes (TPM≥1) between NT—sgRNA and *SCREEM1* locus targeting—sgRNA1 in CRISPR-activated HBEC3-KT cells. NT, non-targeting, n = 3. Red dots, adjusted *p*-value < 10^–6^ and log2 fold change >1; blue dots, adjusted *p*-value < 10^–6^ and log2 fold change <1; black dots, adjusted *p*-value > 10^–6^ and log2 fold change >1; orange dots, >10^–6^ and log2 fold change <1. **(B)** Ranked ordered gene set enrichment analysis of differentially expressed genes between NT-sgRNA and *SCREEM1* locus targeting-sgRNA. NT, non-targeting, n = 3. The EMT pathway is shown. **(C)** Heatmap of EMT-associated genes differentially expressed between NT-sgRNA and *SCREEM1* locus targeting-sgRNA1. NT, non-targeting, n = 3. **(D)** Fold change of key EMT-associated genes differentially expressed between NT-sgRNA and *SCREEM1* locus targeting-sgRNA1. NT, non-targeting, n = 3. NT, non-targeting, n = 3.

Next, we sought to determine whether CRISPR-activation of the *SCREEM locus* led to transcriptome-wide changes. Towards this end we choose the top enriched *SCREEM* sgRNA from the screen (sgRNA1; Supplementary Figure S2A). RNA-sequencing revealed substantial transcriptional reprogramming with 9144 differentially expressed genes following targeting of the *SCREEM* locus (Figure 2A and Supplementary Table S5). Gene Set Enrichment Analysis (GSEA) revealed pathway changes associated with cellular polarity and EMT (Supplementary Figure S2B and Figure 2B). Gene expression of mesenchymal state markers (i.e., *VIM*, *FGF2*, *FN1*, *TGFβ1*) were upregulated while markers of epithelial state (i.e., *EPCAM, CDH1*) were attenuated (Figures 2A, C). Furthermore, master regulators of EMT, i.e., the EMT-TFs *SNAI1*, *ZEB1*, *ZEB2*, and *TWIST1* were also significantly upregulated (Figures 2C, D). Based on these findings, we infer that CRISPR-activation of the *SCREEM* locus produced transcriptome-wide changes consistent with EMT, resulting in CD44 upregulation.

### Activation of the *SCREEM1* locus induces *SNAI1* expression

**FIGURE 3:**
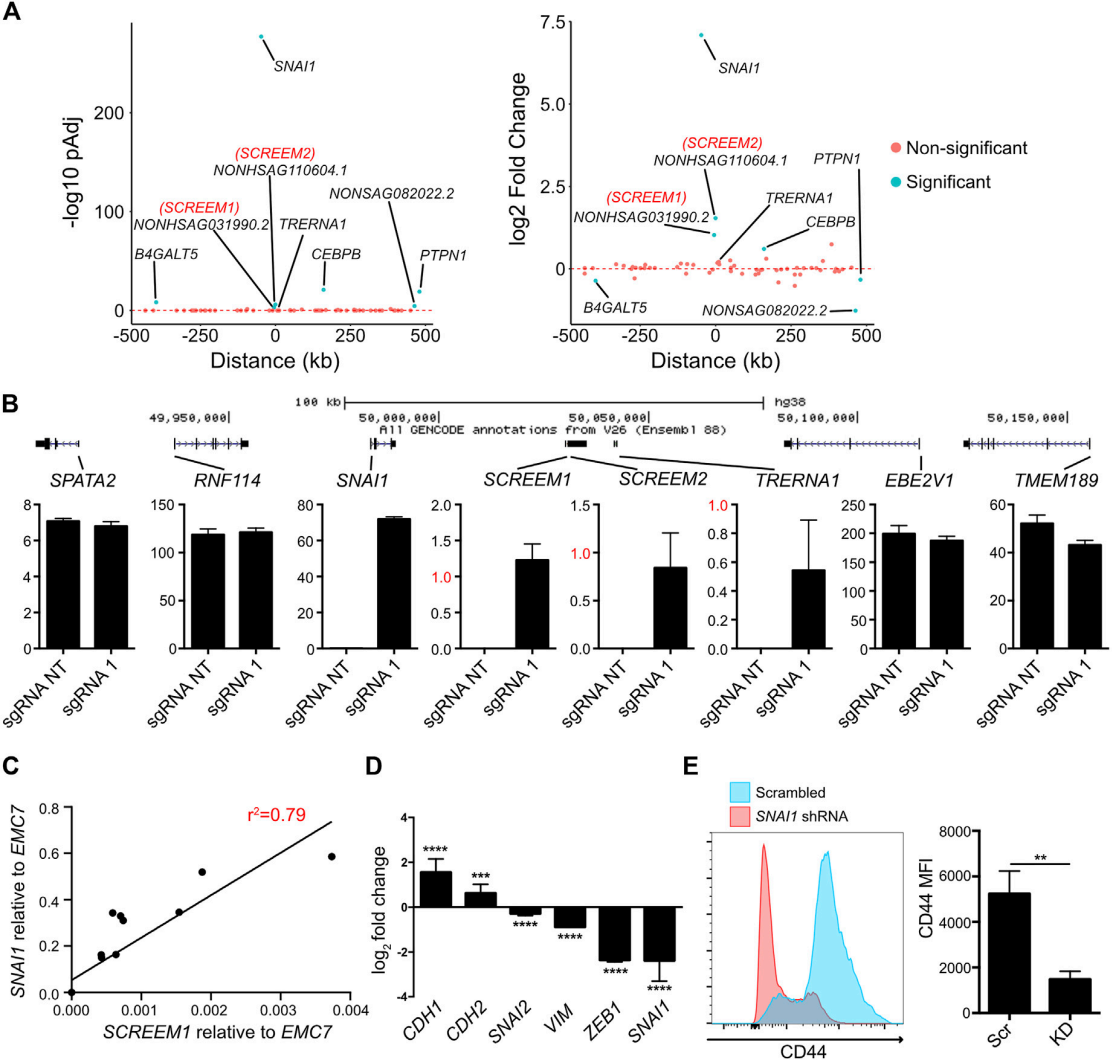
Activation of the *SCREEM1* locus induces *SNAI1* expression. **(A)** Plots showing *p*-value (left) and fold change (right) of genes within 1 mb region surrounding the *SCREEM1* locus from RNA-Seq data described in Figure 2. **(B)** (Top) A scale diagram of *SCREEM1* locus and proximal genes. (Bottom) Expression in TPM values of indicated genes between from RNA-Seq data described in Figure 2. SD; n = 3; NT, non-targeting; sgRNA1, *SCREEM1* locus targeting—sgRNA1. **(C)** Correlation between relative levels of *SCREEM1* (RT-PCR, normalized to *EMC7*) and relative levels of *SNAI1* (RT-PCR, normalized to *EMC7*) following activation of the *SCREEM1* locus. Statistics, simple linear regression. **(D)** RT-PCR analysis of EMT-associated gene expression in *SNAI1*-attenuated HBEC3-KT cells with concomitant CRISPR-activation of *SCREEM1* locus. Plotted are log2 fold changes of delta-delta Ct values relative to scrambled and *EMC7*. All values are mean ± SD with n = 3. Statistics, unpaired *t*-test, ****p < 0.0001; ***p < 0.00. **(E)** Left plot shows representative flow cytometric data of CD44 expression on *SNAI1*-attenuated and Scrambled control HBEC3-KT cells with concomitant CRISPR-activation of *SCREEM1* locus (sgRNA1). Right plot shows mean fluorescent intensity of (MFI) CD44 levels on *SNAI1*-attenuated (KD) and Scrambled control (Scr) HBEC3-KT cells with concomitant CRISPR-activation of *SCREEM1* locus (sgRNA1). All values are mean ± SD with n = 3; Statistics, unpaired *t*-test, **p < 0.01.

One mechanism of lncRNA function is the regulation of proximal genes in *cis* (Gil and Ulitsky, 2020; Statello et al., 2021). To explore the *cis*-regulatory potential of the *SCREEM1* locus we analyzed expression of genes within an ∼ ±500 kb genomic region from the *SCREEM1* TSS. The gene most significantly upregulated following *SCREEM1* activation was the EMT master regulator *SNAI1* (Figure 3A). Although *SCREEM1* was expressed at low levels compared to *SNAI1* (Figure 3B), there was a dosage-dependent expression correlation between the two genes (Figure 3C), further suggesting a *cis*-regulatory mechanism that affects proximal gene expression. SNAI1 is an EMT-TF and a master regulator of EMT (Wang et al., 2013); therefore, to assess whether EMT in this system is driven by SNAI1, we perturbed *SNAI1* expression using shRNAs in *SCREEM1* activated cells (Figure 3D). Here, *SNAI1* attenuation resulted in a partial rescue of the epithelial phenotype; evident as a substantial increase in expression of the epithelial marker *CDH1* and a decrease in expression of mesenchymal markers *CHD2*, *VIM*, *SNAI2* and *ZEB1*(Figure 3D), as well as a significant decrease in surface CD44 expression (Figure 3E). In summary, CRISPRa of the *SCREEM1* locus drives EMT through *SNAI1*, indicating a probable *cis*-regulatory mechanism.

### The *SCREEM* locus contains an enhancer-like element demarcated by multiple eRNAs

**FIGURE 4:**
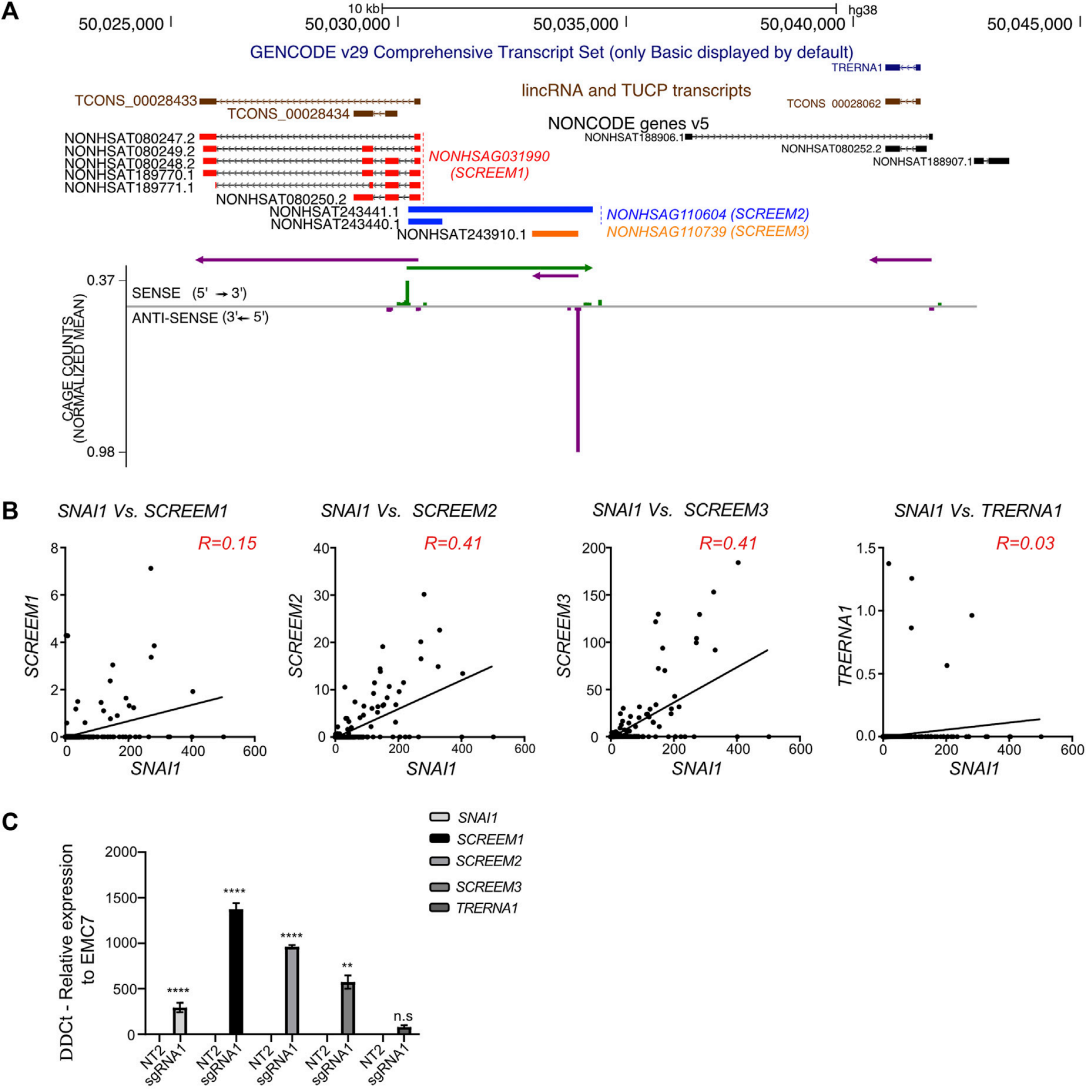
Genomic region proximal to *SCREEM1* contains multiple eRNAs. **(A)** Top, UCSC genome browser view of the *SCREEM1* locus and its proximal genes. On the top the GENCODE v29 (dark blue), lincRNA and TUCP transcripts (brown) and NONCODE (red, *SCREEM1*; blue, *SCREEM2*; orange, *SCREEM3*) annotations are shown. Bottom, plotted normalized CAGE counts mapped to a 5′ cap of *SCREEM1*, *SCREEM2*, and *SCREEM3* (counts shown are from all samples in FANTOM5). **(B)** Normalized CAGE counts mapped to a 5′ cap of *SNAI1*, *SCREEM1*, *SCREEM2*, and *SCREEM3 and TRERNA1* (counts shown were extracted from all samples in FANTOM5). R values were calculated using simple linear regression. **(C)** *SNAI1*, *SCREEM1*, *SCREEM2*, and *SCREEM3* expression in HBEC3-KT cells upon CRISPR-activation of *TCONS_0002834* locus. Plotted are delta-delta Ct values relative to NT and EMC7 from RT-PCR; NT, non-targeting. All values are mean ± SD with n = 3; Statistics, unpaired *t*-test, ****p < 0.0001; ***p < 0.001; **p < 0.01; n.s, not significant.

A closer inspection of the *SCREEM* locus revealed the presence of multiple additional non-coding transcripts annotated in the NONCODE database (Zhao et al., 2016; Fang et al., 2018) *NONHSAG110604* (*SCREEM2*) and *NONHSAG110739* (*SCREEM3*) (Figure 4A). To further examine expression of these transcripts, we investigated the publicly available FANTOM Cap Analysis Gene Expression (CAGE) data, obtained from 1,816 human samples representing a diverse array of cell and tissue types and activation conditions (Lizio et al., 2015; Noguchi et al., 2017; Lizio et al., 2019). We identified CAGE counts for *SCREEM1*, *SCREEM2,* and *SCREEM3* (Figures 4A, B). Furthermore, there was a substantial co-expression correlation between *SNAI1* and each *SCREEM* gene (Figure 4B). Recent reports indicate *TRERNA1* functions as an eRNA in cancer cells; cDNA overexpression or knockdown of *TRERNA1* increases or reduces *SNAI1* expression, respectively (Wu et al., 2017; Song et al., 2019). However, *via* CAGE data analysis we observed little co-expression correlation between *SNAI1* and *TRERNA1* (R = 0.03) (Figure 4B). Moreover, in our genome-wide screen we found no enrichment for sgRNAs targeting the *TRERNA1* TSS in CD44-high population (Supplementary Figure S2C) and *SCREEM1* activation in cells does not induce *TRERNA1* expression but does induce *SCREEM2*, *SCREEM3,* and *SNAI1* (Figure 4C).

**FIGURE 5:**
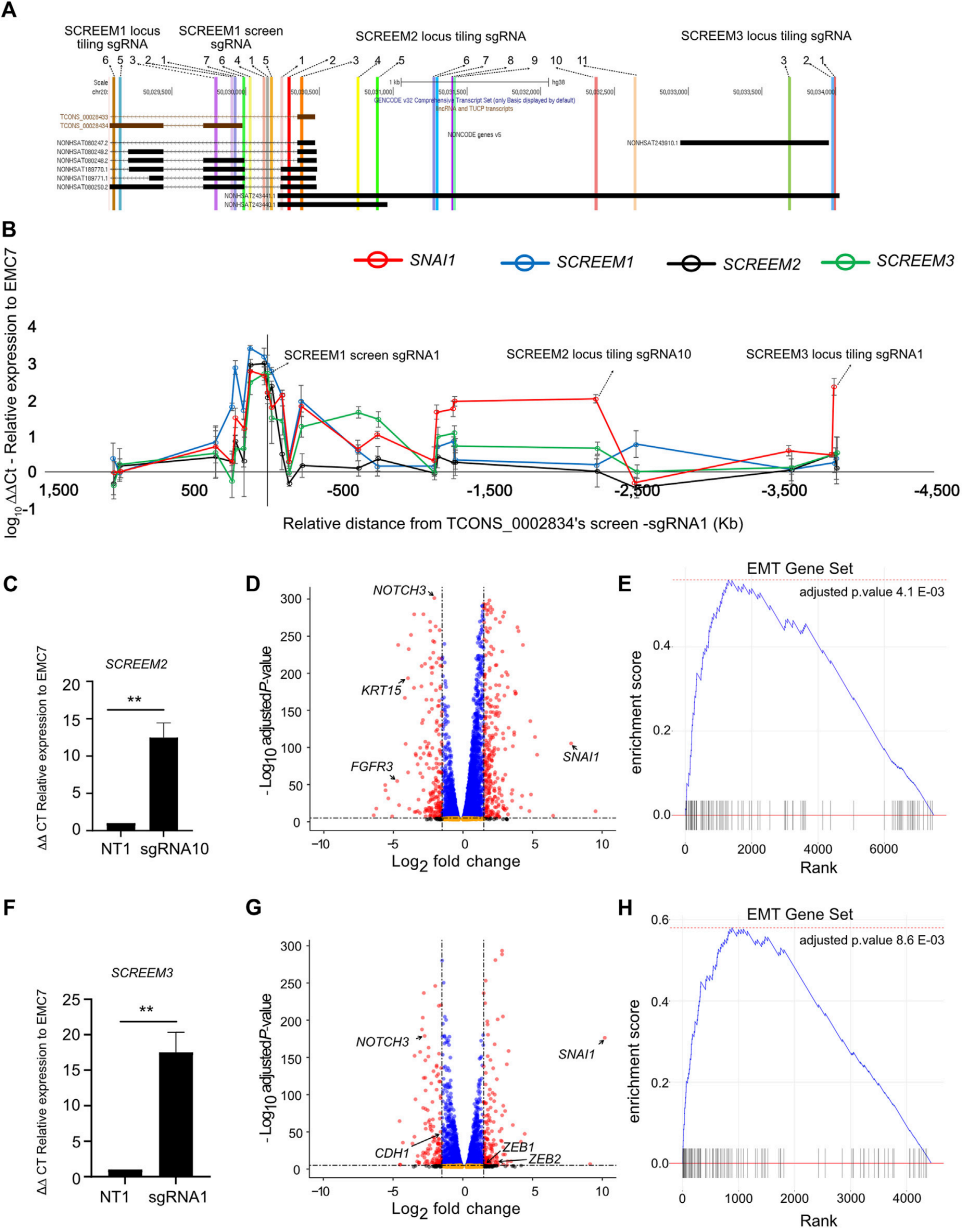
sgRNA tiling reveals an extended enhancer-like region demarcated by eRNA loci which drives activation of *SNAI1*. **(A)** UCSC genome browser view of the *SCREEM1* locus and its proximal genes. The location of different sgRNAs used are indicated**. (B)** RT-PCR analysis of *SNAI1*, *SCREEM1*, *SCREEM2*, and *SCREEM3* expression in HBEC3-KT cells upon CRISPR-activation using sgRNAs are shown. Plotted are log_10_ delta-delta Ct values relative to NT and EMC7; NT, non-targeting. All values are mean ± SD with n = 3; **(C)** RT-PCR analysis showing expression of *SCREEM 2* between NT-sgRNA and *SCREEM 2* locus tiling-sgRNA10. Plotted are delta-delta Ct values relative to NT and EMC7; NT, non-targeting. All values are mean ± SD with n = 3; Stats, unpaired *t*-test, **p < 0.01. **(D)** Volcano plot of expressed genes (TPM≥1) between NT-sgRNA and *SCREEM 2* locus targeting-sgRNA10. NT, non-targeting, n = 3. Red dots, adjusted *p*-value < 10^–6^ and log2 fold change >1; blue dots, adjusted *p*-value < 10^–6^ and log2 fold change <1; black dots, adjusted *p*-value > 10^–6^ and log2 fold change >1; orange dots, >10^–6^ and log2 fold change <1. **(E)** Ranked ordered gene set enrichment analysis of differentially expressed genes shown in **(D)** n = 3. **(F)** RT-PCR analysis showing expression of *SCREEM3* between NT—sgRNA and *SCREEM3* locus tiling—sgRNA1 in CRISPR-activated HBEC3-KT cells. Plotted are delta-delta Ct values relative to NT and EMC7; NT, non-targeting; n = 3; Statistics, unpaired *t*-test, **p < 0.01. **(G)** Volcano plot of expressed genes (TPM≥1) between NT—sgRNA and *SCREEM3* locus tiling—sgRNA1 in CRISPR-activated HBEC3-KT cells. NT, non-targeting, n = 3. Red dots, adjusted *p*-value < 10^–6^ and log2 fold change >1; blue dots, adjusted *p*-value < 10^–6^ and log2 fold change <1; black dots, adjusted *p*-value > 10^–6^ and log2 fold change >1; orange dots, >10^–6^ and log2 fold change <1. **(H)** Ranked ordered gene set enrichment analysis of differentially expressed genes shown in **(G)**.

There are three potential mechanisms by which a lncRNA locus could regulate proximal gene expression: (a) the transcript itself imparts the regulatory capacity (Kotzin et al., 2016), (b) transcription of the region, but not the transcript is required for regulation (Engreitz et al., 2016), and (c) only the underlying DNA elements are required for regulation (Paralkar et al., 2016). To further define the regulatory potential of the locus we tiled sgRNAs across ∼5.5 kb region encompassing the 3’ region of *SCREEM1* and the TSS of *SCREEM3* (Figure 5A). Stable CRISPRa of HBEC3-KT cells with individual sgRNAs were established and expression of *SNAI1, SCREEM1*, *SCREEM2,* and *SCREEM3* were evaluated by RT-PCR. There was a strong correlation between induction of non-coding transcripts and expression of *SNAI1*, i.e., only sgRNAs that induced lncRNA expression were able to induce expression of *SNAI1* (Figure 5B and Supplementary Figure S3A). Given that we were unable to separate the enhancer-like potential of the underlying DNA elements and transcription through the locus, this could suggest that transcription at the *SCREEM* loci may be essential for *SNAI1* induction. Enhancers can interact with promoters in enhancer-promoter loops; thus, it is possible that dCas9-SAM transcriptional activators bound to the regulatory element could simultaneously act directly on the *SNAI1* promoter. However, sgRNAs directly targeting the *SNAI1* promoter did not induce expression of non-coding transcripts within the locus, suggesting that any regulatory function was unidirectional and likely not simply an artefact of the SAM system driven by enhancer-promoter loops (Supplementary Figure S4A).

To understand whether CRISPRa of other regions of the *SCREEM* locus had a similar transcriptome-wide effect we performed RNA-seq analysis of two additional CRISPRa cell lines – *SCREEM2* tiling-sgRNA10 (Figure 5C and Supplementary Table S6) and *SCREEM3* TSS targeting-sgRNA1 (Figure 5F and Supplementary Table S7). Here we confirmed a robust EMT gene signature (Figures 5D, E, G, H and Supplementary Figures S5A–D). In both the cell lines, expression of mesenchymal state markers – *ZEB1*, *ZEB2*, and *VIM*—were upregulated, whereas markers of epithelial state – *NOTCH3*, *EPCAM*, and *CDH1*—were attenuated (Supplementary Figures S5B, D). Again, *SNAI1* was the most significantly upregulated EMT-TF (Figures 5D, G), providing further evidence that EMT induced through activation of the lncRNA cluster correlated with increased *SNAI1* expression.

Inspection of sequencing reads aligned at the lncRNA cluster revealed the presence of bi-directional transcripts proximal to each sgRNA binding site (Supplementary Figure S6A). Interestingly, while all three sgRNAs robustly induced *SNAI1* expression, they each elicited different transcripts within the targeted region, indicating that no single transcript is essential for the regulatory function of the locus. We also saw no evidence for active transcription of the *TRERNA1* locus. Collectively, these data suggest that this genomic region is a *SNAI1* regulatory element composed of multiple redundant enhancer-like elements demarcated by eRNAs. Our data also suggest that although transcription of the locus may be important, the transcripts themselves are likely dispensable.

### The eRNA transcripts are dispensable for activation of *SNAI1*

Subcellular fractionation demonstrated that *SCREEM1* transcripts were localized across cytoplasm, nucleoplasm and chromatin and so could potentially regulate *SNAI1* expression by either a *cis-* or *trans*-based mechanism (Supplementary Figure S7A). In contrast, transcripts for *SCREEM2* and *SCREEM3* were concentrated in the nucleoplasm and chromatin fractions (Supplementary Figure S7A). To directly test whether the eRNA transcripts were required for the regulation of *SNAI1* expression*,* we used shRNAs to separately knockdown all three *SCREEM* transcripts in *SCREEM* locus CRISPRa cells. In no instance did eRNA knockdown attenuate *SNAI1* upregulation (Supplementary Figure S7B), supporting our interpretation of the tiling data. Similarly, stable lentiviral-directed overexpression of *SCREEM2* and *SCREEM3* did not induce *SNAI1* expression (Supplementary Figure S7C). Unlike *SCREEM2* and *SCREEM3, SCREEM1* is a multi-exonic transcript and we were unable to resolve the precise sequence and so we were unable to perform overexpression of the *SCREEM1* transcript in cells. Non-etheless, in combination with the tiling data presented above, these data together indicate that either the eRNA transcripts are likely dispensable for activation of *SNAI1,* or that there is redundancy between them.

### *SCREEM* eRNAs and *SNAI1* are actively transcribed from a super-enhancer in monocytes

**FIGURE 6:**
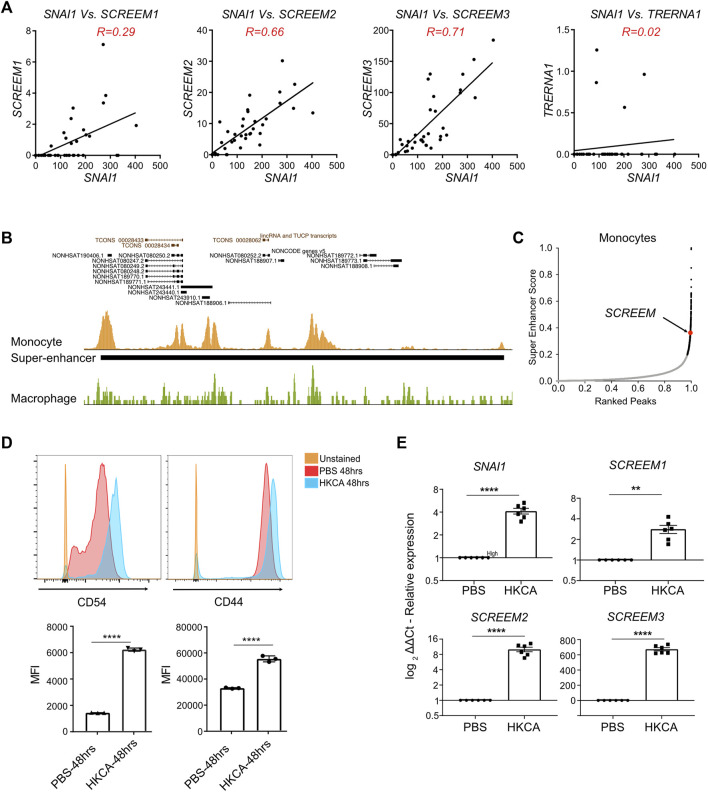
Monocytes stimulated with C. albicans express *SNAI1* enhancer eRNAs and *SNAI1.* **(A)** Normalized CAGE counts mapped to the 5′ cap of *SNAI1*, *SCREEM1*, *SCREEM2*, and *SCREEM3,* and *TRERNA1* (counts shown were extracted from all monocyte samples in FANTOM5). R values were calculated using simple linear regression. **(B)** A UCSC genome browser view of the *SCREEM* loci and proximal genes is shown on top. Peaks of H3K27ac from ChIP-Seq data from monocytes (gold) a macrophages (green) is show below. The solid back bar shows the position of a region annotated as a super-enhancer by the HOMER pipeline. **(C)** Plots showing monocyte enhancers ranked by super-enhancer score. Super-enhancers (as defined by where slope is greater than 1) are indicated by black dots. The position of the *SCREEM* super-enhancer is annotated in red. **(D)** On top are representative flow cytometric plots of CD54 (left) and CD44 (right) expression on monocytes exposed to HKCA or PBS for 48 h. Below shows quantification of mean fluorescence intensity (MFI) of CD54 and CD44 staining. Plotted values are mean ± SD with n = 3, biological triplicates. Statistics, unpaired *t*-test, ****p < 0.0001. **(E)** RT-PCR analysis of *SCREEM1*, *SCREEM2*, and *SCREEM3* and *SNAI1* expression in monocytes exposed to HKCA or PBS for 48 h. Plotted are delta-delta Ct values relative to mock and EMC7; NT, Non-targeting. All values are mean ± SD with n = 6, independent donors. Statistics, unpaired *t*-test, ****p < 0.0001, **p < 0.01.

The SAM experiments in bronchial epithelial cells revealed that activation of the *SCREEM* locus resulted in a potent SNAI1-mediated EMT. However, we could find no evidence that *SCREEM* locus was active in either healthy or diseased airway epithelium. To identify relevant tissues in which *SCREEM* was active we returned to the FANTOM5 CAGE datasets (Lizio et al., 2015; Noguchi et al., 2017; Lizio et al., 2019). From this data it was evident that expression of these eRNAs was restricted to monocytes, where we saw a strong correlation with *SNAI1* expression under various activation conditions. We found co-expression correlations between *SNAI1* and *SCREEM1* (R = 0.29)*, SNAI1* and *SCREEM2* (R = 0.66), and *SNAI1* and *SCREEM3* (R = 0.71) specifically in monocytes (Figure 6A and Supplementary Figure S8A). These results suggest that this region may represent a *SNAI1* enhancer in monocytes. To test this hypothesis, we analyzed BLUEPRINT ChIP-Seq data for evidence of enhancer activity. Interestingly, the enhancer defining histone modification, H3K27ac, was highly enriched across the *SNAI1 cis*-regulatory region in monocytes, but not in monocyte-derived macrophages (Figure 6B, Supplementary Figure S8B, and Supplementary Table S8). Moreover, HOMER analysis indicated that this region was a possible super-enhancer, suggesting a potentially important role in monocytes (Figures 6B, C and Supplementary Figure S8B).

To confirm whether monocytes actively co-transcribed these *cis*-regulatory eRNAs and *SNAI1*, we isolated monocytes from PBMCs and stimulated them with heat-killed *Candida albicans* (HKCA). Here we observed CD54 (ICAM-1) a marker of activated monocytes and CD44 to be upregulated after stimulation with HKCA (Figure 6D). Furthermore, *SCREEM1*, *SCREEM2*, *SCREEM3* and *SNAI1* were all significantly upregulated (Figure 6E). Together, these findings further support the notion that this *cis*–regulatory region demarcated by eRNAs regulates *SNAI1* expression in monocytes. Thereby taking this account, we termed the eRNAs bearing the enhancer loci as *SCREEM* (*SNAI1 cis*-regulatory eRNAs expressed in monocytes) and the individual transcript locus *NONHSAG031990*, *NONHSAG110604*, and *NONHSAG110739* as *SCREEM1*, *SCREEM2*, and *SCREEM3* respectively. Understanding the role of the *SCREEM* locus and SNAI1 function in monocytes could reveal novel aspect of innate immunity.

## Discussion

The human genome contains a vast array of uncharacterized ncRNA genes such as lncRNAs. Although these genes have potentially important roles in human health and disease, the function of most is completely unknown. Contributing to this lack of knowledge is our current inability to reliably predict lncRNA function based on sequence alone. Thus, these genes must be interrogated with direct experimentation to determine their biological potential. Genome-wide screens offer an attractive approach for functional assessment of the lncRNA landscape at scale. However, lncRNAs are typically expressed in a highly tissue specific manner (Jiang et al., 2016), and a limited number are expressed in any given cell type, thereby restricting our ability to test their function. Activation screens overcome this limitation by allowing interrogation of lncRNA function outside of their normal tissues or cell type. However, a caveat of this approach is that any uncovered functions must be then validated in relevant cell types. To capture novel functional lncRNA loci we performed a CRISPRa screen in the context of EMT. EMT was chosen as it is well characterized fundamental molecular process coordinated by a complex transcriptional program, making it an ideal candidate for regulation by lncRNAs (Xu et al., 2016; Cheng et al., 2019; Gugnoni and Ciarrocchi, 2019).

By implementing a novel screening strategy based on surface CD44 expression, we identified numerous gRNAs that were enriched in either epithelial-like (CD44-low) or mesenchymal-like (CD44-high) cells, suggesting that their target lncRNAs had potential to either activate or attenuate EMT, respectively. A more detailed investigation of the *SCREEM* locus, revealed multiple eRNA-like transcripts—*SCREEM1, SCREEM2* and *SCREEM3*— that marked a novel *cis*-regulatory locus that coordinated *SNAI1* activation. We found that CRISPRa mediated targeting of *SCREEM1* TSS led to bi-directional transcriptional activity across an ∼5 kb region and activation of the *SNAI1* gene, which lies approximately 50 Kb upstream. SNAI1, an EMT-TF, is a member of the SNAI1 family of zinc-finger TF that usually functions as a transcriptional repressor of epithelial genes (Wu and Zhou, 2010; Serrano-Gomez et al., 2016; Stemmler et al., 2019). RNA-Seq analysis of *SCREEM* locus-activated cells revealed a robust transcriptional reprogramming consistent with EMT, which was reversed on *SNAI1* knockdown. In contrast, shRNA mediated knockdown of all three *SCREEM* transcripts*,* did not attenuate *SNAI1* expression. However, these knockdowns were incomplete and so the remaining eRNA transcripts may have been sufficient for regulation. In addition, it is possible that the transcripts may have only been required to initiate *SNAI1* expression and not its maintenance, as has been suggested for other eRNAs (Li et al., 2016a; Rahman et al., 2017). Conversely, lentiviral overexpression of these transcripts did not induce *SNAI1* expression, although this is not necessarily surprising as only some eRNAs have been shown to function in *trans* (Feng et al., 2006). Finally, in the *SCREEM* loci tiling experiments, although different sgRNAs were able to drive *SNAI* expression, they induced different *SCREEM* transcripts. All together these findings imply that the DNA or transcription within this locus, but not the individual eRNA transcripts themselves, are likely important for *SNAI1* activation. Such mechanisms of proximal gene regulation in *cis* that are independent of the associated non-coding transcripts have been shown for loci such as *Bendr* (Engreitz et al., 2016), *Rroid* (Mowel et al., 2017)and *Lockd* (Paralkar et al., 2016).

An alternative explanation is that SAM transcriptional activators recruited to the *SCREEM* locus could have directly activated the *SNAI1* promoter, potentially through DNA looping. However, in tiling experiments, gRNAs that failed to activate the expression of lncRNAs within the locus region also failed to activate *SNAI1*. In addition, sgRNAs directly targeting the *SNAI1* promoter also did not induce expression of non-coding transcripts within the locus, suggesting that any regulatory function was unidirectional and likely not simply an artefact of the SAM system driven by enhancer-promoter loops. This observation fits with other eRNA studies (Carullo et al., 2020).

Although our data showed that activation of the *SCREEM* loci could drive robust SNAI1-mediated EMT in lung epithelial cells, we found no evidence that these loci were active in the lung epithelium under normal physiologic conditions. This finding highlights the limitations of activations screens in which lncRNAs are studied outside of their natural context. However, our goal was not to identify genes important in lung biology, but rather to exploit the process of EMT to uncover ncRNAs with novel functions. Through analysis of a large CAGE dataset we observed, *SCREEM1, SCREEM2, SCREEM3,* and *SNAI1* were co-expressed in activated primary human monocytes. Interestingly, these transcripts appear to demarcate a super-enhancer that is present in monocytes but not in monocyte derived macrophages. Therefore, we termed the eRNAs bearing the enhancer loci as *SCREEM*1 (*SNAI1 cis*-regulatory eRNAs expressed in monocytes), *SCREEM2*, and *SCREEM3*.

Our findings raise the question as to the role of the *SCREEM* loci and SNAI1 in monocytes. Super-enhancers have been described as potent cell-type-specific regulatory elements that frequently control expression of genes specifying and maintaining cell identity. Does this imply an important role for SNAI1 in monocytes? While SNAI1 is chiefly thought of as a master regulator of EMT, emerging evidence demonstrates function outside of EMT, i.e., upregulated SNAI1 expression in fibroblasts and neoplastic mesenchyme cells and their influence on macrophages found at the site of inflammation (Wu and Zhou, 2010; Wang et al., 2013; Stemmler et al., 2019). Moreover, given that other EMT master regulators have been implicated in immune cell function, a potential role for SNAI1 in immunity is not without merit. *SNAI1* is known to be expressed in human monocytes but is substantially downregulated during monocyte-to-macrophage differentiation (Saeed et al., 2014). Although the significance of this is unknown, experiments in the monocytic THP-1 cell line have suggested that SNAI1 may play a role in macrophage polarization (Zhang et al., 2016). However, to our knowledge, this function has never been confirmed in primary mouse or human cells. Based on our studies in epithelial cells and primary human monocytes, we speculate that SNAI1 may help to regulate CD44 expression, potentially to coordinate monocyte homing. As we have been unable to successfully knockdown SNAI1 in monocytes we have not been able to test this hypothesis. In contrast to the healthy immune system, SNAI1 has been shown to contribute to pathogenesis in acute monocytic leukemia (AML) (Carmichael et al., 2020). In future studies it will be exciting to explore the function of the *SCREEM*-*SNAI1* axis in monocyte biology and potentially in AML. Lastly, our study demonstrates the value of genome-wide CRISPRa screens in identifying functional ncRNA loci with unique biological roles.

## Medthods

### Cell lines

HBEC3–KT and HEK293T cells were obtained from ATCC and were tested for *mycoplasma* contamination quarterly. HBEC3-KT cells were validated using short tandem repeat profiling at ATCC.

### HBEC3–KT cultures

*Mycoplasma* tested HBEC3–KT were cultured at 37 °C in a humidified atmosphere with 5% CO2 in culture ware pre-coated with 0.1% pig skin gelatin (Sigma Aldrich, Cat# G1890). Briefly, when cultures were about 70%–80% confluent, spent medium was removed and discarded. Dulbecco’s Phosphate Buffered Saline, was used to rinse off dead cells. 1 mL of Trypsin-EDTA Primary Cells (ATCC)/25 cm^2^ was added and incubated at 37 °C for 4–6 min (until 90% of the cells have detached). 2% FBS in DPBS at 1 mL/25 cm2 was added to neutralize the trypsin. Cell suspensions were centrifuged at 1000rpm for 5 min at room temperature. Viable cells resuspended in Airway Epithelial Cell Basal Medium (ATCC) supplemented with Bronchial Epithelial Cell Growth Kit (ATCC) and seeded at 4.0 x 10^3^ to 6.0 × 10^3^ cells/cm2 in flasks pre-coated with 0.1% pig skin gelatin.

### TGFβ1 treatment on HBEC3–KT cultures

*Mycoplasma* tested HBEC3–KT were seeded (50,000 cells/cm^2^) in complete Airway Epithelial Cell Basal Medium supplemented either with 10 ng/ml of TGFβ1 (R&D) or PBS. Cells were harvested 72 h s after seeding for various experimentations.

### Plasmids, lentiviral vectors, and production of lentiviruses

All plasmids are listed in Supplementary Table S9, sgRNAs cloned (Supplementary Table S10) into expression lentivector, shRNAs sequences (Supplementary Table S10) cloned into shRNA expression lentivector, and cDNA sequences (Supplementary Table S11) cloned into expression lentivector were used in the generation of lentiviral particles. Lentiviruses were harvested using standard procedures in *Mycoplasma* tested HEK293T cells. Briefly, 1 day before transfection, 4.5 × 10^6^ 293T cells were plated in a 10 cm dish with 10 mL of DMEM-complete media (DMEM+10% FBS+1% pen-strep+1% Sodium pyruvate). A mixture of the three packaging plasmids, pLP1, pLP2, and pLP/VSVG (ViraPower™ Lentiviral Packaging Mix) and lentiviral expression vector were transfected into HEK293T cells using Polyethylenimine (PEI) transfection reagent (Polysciences) in DMEM complete media free of antibiotics. 16–18 h post transfection spend media was replaced by DMEM-complete media. 48 h after transfection, the virus supernatants were harvested, filtered using 0.45um low protein binding membrane (PES), and precipitated with Polyethylene glycol (PEG) 6000 for synthesis (CAS 25322-68-3, pH 5 – 7). The final precipitated viral particles were mixed with polybrene (8 μg/ml) and transduced into *Mycoplasma* tested HBEC3–KT.

### Density gradient separation of live and dead cells

The harvested HBEC3–KT cells were resuspended in pre-warmed Airway Epithelial Cell Basal Medium (ATCC) (10 million/7 mL). This cell suspension was then slowly added to a layer of Lymphoprep (Stemcell Technologies) in the bottom of the Falcon tube (3.5 mL for 7 mL cell suspension) and spun at 1300rpm for 30 min at room temperature with low acceleration and no brake. The top clear media was carefully aspirated, and layer of cells (1–2 mL/10^6^ cells) was harvested using a P1000. The harvested cells were suspended in Airway Epithelial Cell Basal Medium (7 mL for 10 million cells) and pelleted (1300 rpm for 10 min at room temperature). Supernatant was discarded, the cells were resuspended in antibody staining buffer (0.5 mM EDTA and complete-Airway Epithelial Cell Basal Medium).

### Flow cytometry staining, cell sorting, and analysis

Single-cell suspensions were stained with Live/Dead Ghost (VWR) to exclude non-viable cells. Subsequently, the washed and pelleted cells were stained with CD44 or CD54 with indicated fluorochrome-conjugated antibodies. All flow cytometry analysis and cell-sorting procedures were done at The Jackson Laboratory Flow Cytometry and Cell Sorting Facility using BD LSRII cell analyzers and a BD FACSAria II sorter, running FACSDiva software (BD Biosciences). FlowJo software (version 10 TreeStar) was used for data analysis and graphic rendering. All fluorochrome-conjugated antibodies used are listed in Supplementary Table S12.

### CRISPRa screen (pooled library amplification, lentivirus transductions, antibiotic selection, EMT induction, flow sorting, and sequencing)

The screen was performed as previously described (Joung et al., 2017b) with modifications. Briefly, 50–100 ng/μL of the sgRNA library (Addgene, pooled Library #1000000106) was electroporated (total of 10 electroporation,1-electroporation/10,000 sgRNAs in the library) using Endura ElectroCompetent cells according to the manufacturer’s directions. Two mL of electroporated cells were platted on to large agar plates (Teknova) and after 14hrs of incubation, the electroporation efficiency (>100 colonies per sgRNA in the library) was calculated by counting the number of colonies on the 10,000-fold dilution plate. Colonies were harvested from the LB agar plates and the amplified plasmid was purified using an endotoxin-free plasmid purification (Qiagen). To determine distribution of the amplified pooled library, the spectrophotometer quantified plasmid product was sequenced using primers (listed in Supplementary Material) in Illumina MiSeq (80 cycles of read 1 (forward) and eight cycles of index 1 with a 5% PhiX control to improve library diversity in order to cover >100 reads per sgRNA in the library). Next, the lentiviruses were produced using the amplified pooled library using methods described above. Briefly, four T225 flasks (seeded at 1.8x10^7^ cells per flask) were transfected with the pooled library constructs and lenti-viral packing plasmids. Forty-8 hrs later, the lentiviral particles were harvested, filtered, concentrated, and stored at −80C. HBEC3-KT zeocin resistance was determined using kill curve (50ug/ml). Therefore, using the resistance data, the lentiviral titer was calculated in HBEC3-KT cells (stably expressing dcas9-vp64 and P65) using CellTiter Glo (Promega, PR-G7570) according to the manufacturer’s protocol. 330million HBEC3-KT cells were transduced (stably expressing dcas9-vp64 and P65) with pooled sgRNA contained lentiviral particles at a MOI of 0.3 (1000X representation of the library in surviving cells). The transduced cells were selected with Zeocin (50ug/ml) for 14-day (maintaining a 500X coverage per passage, i.e. 50 million cells). The SAM expressing HBEC3-KT cells (Zeocin [sgRNA-hsf1-lib; (50ug/ml)], Hygromycin [p65(10ug/ml)], and blasticidin dcas9-vp64(10ug/ml)] were then treated with 10 ng/ml of TGFβ1 (R&D) for 72hrs. Subsequently, the cells were harvested and processed for CD44 staining as described (see section – Ficoll separation of live and dead cells, and Flow cytometry staining, cell sorting, and analysis). Note, for cell sorting using BD FACSAria II sorter, 120-micron tip was used to allow sorting of large mesenchymal-like cells. We sorted ∼50X representation of the library from CD44-low (bottom 10% of the peak) and CD44-high (top 10% of the peak) cells. Genomic DNA was harvested from sorted populations using the Quick-DNA plus (Zymo Research) according to the manufacturer’s protocol. To determine distribution of the sgRNA, the illumina libraries (primers listed in Supplementary Material) were normalized and pooled. Quantification of library pool was performed using real-time qPCR (KAPA and Thermo Fisher). The final library pool was normalized to 2 nM. The pool was then denatured and loaded on the illumina sequencer as per the manufacturer’s instructions (Illumina). PhiX was spiked in at 5%. Sequencing was performed on Illumina NextSeq platform generating single end reads of 80bp (80 cycles of read 1 (forward) and eight cycles of index 1. 5% PhiX control was used to improve library diversity to cover >50 reads per sgRNA in the library.

### Analysis of CRISPRa library

The sgRNA library was obtained from Joung et al. (Joung et al., 2017a). Sequencing was performed on Illumina platform generating paired end reads of 80 bps. Fragments were trimmed using trim galore software (https://github.com/FelixKrueger/TrimGalore) and reads with quality <20 were filtered out. Guide counts were calculated using count_spacers.py script from Joung et al. (Joung et al., 2017b). The significant genes from the screen were identified with MAGeCK software (Li et al., 2014). Any gRNAs targeting more than one lncRNA were excluded from analysis.

### Subcellular RNA fractionation

Measurement of the abundance of chromatin, nucleoplasm, and cytoplasmic RNA was performed as described previously (Mayer and Churchman, 2017) and modified for HBEC3-KT cells. In short, 5x10^6^ HBEC3-KT cells were pelleted (1500 rpm, 5 min) and washed with sterile 1X PBS. To this pellet, 380ul of Hypotonic Lysis Buffer supplemented with 100U of SUPERase-In Rnase Inhibitor (Life Technologies) was added. Hypotonic Lysis Buffer: 50 mM TRIS-HCl pH7.4, 50 mM NaCl, 3mM MgCl2, 0.5% NP-40, 10% Glycerol. Resuspend pellet was vortexed for 30 s, incubated on ice 30 min and vortexed for additional 30 s before pelleting (1000g, 5 min, 4C). Supernatant was collected as the cytoplasmic fraction and the pellet was collected as nuclear fraction. To the cytoplasmic fraction 1 mL of RPS buffer was added and vortexed for 30 s and stored at −20C for at least 1 hour (not more than one or 2 days). RPS buffer: 9.5 mL ethanol (200-proof) + 0.5 mL Sodium Acetate (3M pH 5.6). To lyse the nuclear membranes, 380ul of Modified Wuarin-Schibler buffer supplemented with 100U of SUPERase-In Rnase Inhibitor (Life Technologies) was added to the nuclear fraction. Modified Wuarin-Schibler buffer: 10 mM TRIS-HCl pH7.4, 0.3M NaCl, 4 mM EDTA, 1M Urea, 1% NP-40. Resuspend pellet was vortexed for 20 s and incubated on ice for 10–12 min and vortexed for additional 30 s before pelleted (1000g, 5 min, 4C). Supernatant was collected as the nucleoplasm fraction and the pellet was collected as chromatin fraction. To the chromatin fraction 1 mL of TRIZOL was added and vortexed for 30 s and stored at −20C for at least 1 hour (not more than one or 2 days). To the nucleoplasmic fraction, 1 mL RPS buffer was added, vortexed, and store at −20C. Then, both the cytoplasmic and nucleoplasmic fraction were vortexed for 30 s pelleted (15 min, 21000g, 4C). Subsequently, the pelleted fractions were washed with ice cold 70% ethanol, vortexed for 30 s and pelleted (5 min, 18000g, 4C). Finally, ethanol was aspirated, and the pellet was air dried for 3–5 min. Then, 1 mL of TRIZOL was added and vortexed for 30 s. Now, to all the fractions in 1 mL of TRIZOL, 10ul of 0.5M EDTA was added, heated at 65C for 10 min, cooled to room temp for 10 min. To this mixture, 200ul of chloroform was added, vortexed and pelleted (21000 g × 10 min at room temp). The clear aqueous (top) layer was harvested and one volume of equal amount of 70% ethanol (400-500ul) was added. Finally, this mixture was loaded to Rneasy column and RNA was extracted according to manufacture protocol. Note, during qRT-PCR analysis for the subcellular runs, either the CT value of the cytoplasmic fraction or the CT value of the chromatin fraction for the transcript of interest was used for normalization (instead of normalizing using housekeeping transcript CT values).

### RNA extraction, cDNA synthesis and quantitative RT-PCR

Total RNA was extracted using RLT buffer supplemented with Beta-mercaptoethanol according to the manufacturer’s instructions (Qiagen). Isolated RNA was quantified by spectrophotometry, and RNA concentrations were normalized. cDNA was synthesized using SuperScript III Reverse Transcriptase (ThermoFisher Scientific) according to the manufacturer’s instructions. Resulting cDNA was analyzed by SYBR Green (KAPA SYBR Fast, KAPABiosystems) using indicated primers. Primer sequences are listed in Supplementary Table S12. All reactions were performed in triplicates using ViiA7 Real-Time PCR instrument (ThermoFischer Scientific).

### Primary monocytes isolation, culture, and activation

Blood was collected in accordance with The Jackson Laboratory for genomic medicine Institutional Review Board. Donor details are available in Supplementary Table S14. Peripheral blood mononuclear cells (PBMC) were isolated by density gradient centrifugation using Lymphoprep (Stemcell Technologies) according to manufactures protocol. The isolated cells were incubated with CD14 MicroBeads (miltenyibiotec) and CD14^+^ monocytes were isolated based on manufacturer’s instructions. CD14^+^ monocytes were cultured in RPMI 1640 medium (Fischer) supplemented with 1% penicillin/streptomycin (GIBCO), heat inactivated 10% fetal bovine serum (FBS, Seradigm), 1% sodium pyruvate (GIBCO), 0.05mM B-mercaptoethanol (GIBCO) and hMCSF (10ng/ml) (R&D Systems). For activation of monocytes, 5x10^5^ CD14^+^ monocytes were incubated with 5x10^7^ HKCA (InvivoGen) for 48hrs. Cells were then harvested, washed with 1X PBS (3 times), pelleted (1500rpm, 5 min) and processed for either flow cytometry analysis or RNA extraction.

### RNA extraction, sequencing, and analysis

#### RNA-seq analysis

Total RNA was extracted using RLT buffer supplemented with β-mercaptoethanol (Qiagen) according to the manufacturer’s instructions. Isolated RNA was quantified by spectrophotometry, and RNA concentrations were normalized. Sequencing was performed on Illumina platform generating paired end reads of 75 bps for the dataset of sgRNA SCREEM1 (polyA enrichment) and 150 bps for the dataset of sgRNAs SCREEM2 and SCREEM3 (ribo depletion). Fragments were trimmed using trim galore software (Krueger et al., 2021) and reads with quality <20 were filtered out. Fragments were quasi-mapped to the human transcriptome either with Gencode annotation or the combined Gencode (Frankish et al., 2021) and Non-code annotations (Zhao et al., 2021) as previously described (Uthaya Kumar et al., 2022). using salmon (version 0.7.2) (Patro et al., 2017). Gene level differential expression analysis was performed using DESeq2 package in R (Love et al., 2014). The cut off was for differentially expressed (DE) genes was set at < 0.001 adj. *p*-value. DE genes where then used for different analysis and plotting using R studio. Volcano plots using EnhancedVolcano, GSEA using clusterProfiler (Yu et al., 2012), and heatmaps using pheatmaps in R (Kolde, 2019). EMT-associated gene list was curated from various database and used as reference for EMT-heatmaps (Supplementary Table S13).

#### ChIP-seq and super-enhancer analysis

ChIP-seq dataset from Blueprint EGAD00001001011 (Monocyte - EGAF00000604457 and Macrophage - EGAF00000284341) was used for super-enhancer identification. Fragments were trimmed using trim galore software (Krueger et al., 2021) and reads with quality <20 were filtered out. Reads were mapped to hg38 genome using BWA-MEM aligner (Li, 2013). MACS2 tool was used for peak calling (Feng et al., 2012) and *findPeaks* tool from Homer suite with “*-style super”* option was used for super-enhancer identification (Heinz et al., 2010).

#### CAGE analysis

CAGE counts across all samples in FANTOM were extracted using ZENBU (Severin et al., 2014) browser for *SNAI1*, *SCREEM1*, *SCREEM2*, *SCREEM3*, and *TRERNA1*.

## Data Availability

All sequencing data presented in this publication have been deposited in NCBI’s Gene Expression Omnibus and are accessible through GEO Series accession number GSE223684 (https://www.ncbi.nlm.nih.gov/geo/query/acc.cgi?acc=GSE223684).

## Supplementary material

Supplementary Figure S1 CRISPRa screen identifies candidate lncRNA loci in EMT regulation. **(A)** Plasmid single guide RNA (sgRNA) library counts following bacterial amplification, indicating high quality capture of sgRNAs in the library post amplification. **(B)** Plasmid sgRNA library ranked based on amplification counts, a skew ratio 2.3 indicates good representation of individual sgRNAs in the amplified library. **(C)** Library-containing HBEC3-KT cells treated with TGFβ1 (10ng/ul) for 72hrs before cell sorting based on cell surface expression of CD44. Bottom 10% of CD44 expressing cells (CD44-low) and top 10% CD44 expressing cells (CD44-high) were separating by cell sorting. Top, depicts pre-sort, bottom depicts post-sort n = 3. **(D)** Counts representing three sgRNA in CD44-low and CD44 high fractions plotted from six candidate lncRNA loci identified from the CRISPRa screen, n = 3. **(E)** RT-PCR showing transcriptional activation of candidate lncRNAs in HBEC3-KT cells upon SAM targeting of indicted loci. Plotted are delta-delta Ct values relative to NT1 and EMC7 (housekeeping gene): NT, non-targeting. All values are mean ± SD with n = 3. **(F)** Representative flow cytometry plots showing analysis of CD44 expression in HBEC3-KT cells upon CRISPR-activation of candidate lncRNA loci. NT, non-targeting. n = 2 or 3 sgRNAs per gene and shown is one representative of 3 replicates.

Supplementary Figure S2 Activation of the SCREEM1 locus elicits transcriptional changes associated with EMT. **(A)** sgRNA counts plotted for all sgRNAs targeting SCREEM1 TSS from the screen. Top 3 sgRNAs chosen for further validation experiments are highlighted in red. n = 3. **(B)** RNA-seq analysis of CRISPR-activation of SCREEM1 locus (sgRNA1) in HBEC3-KT cells. Ranked ordered gene set enrichment analysis of differentially expressed genes showing top significant pathways, NT, non-targeting, n = 3. **(C)** sgRNA counts plotted for all sgRNAs targeting TRERNA1 TSS from the screen.

Supplementary Figure S3 sgRNA tiling reveals an extended enhancer-like region demarcated by eRNA loci which drives activation of SNAI1. **(A)** Dot plot showing RT-PCR analysis of the sgRNA tiling experiment **(**from Figure 5B **)** showing correlation between expression of SNAI1 and indicated SCREEM eRNAs. n = 3. R values were calculated using simple linear regression.

Supplementary Figure S4 sgRNAs targeting SNAI1 promoter significantly induced expression of SNAI1 but did not impact expression of neighboring eRNAs. RT-PCR analysis of SNAI1, SCREEM1, SCREEM2, and SCREEM3 expression in HBEC3-KT cells upon CRISPR-activation of the SCREEM1 locus or the SNAI1 locus. Plotted are delta-delta Ct values relative to NT and EMC7; NT, Non-targeting. All values are mean ± SD with n = 3. Statistics, unpaired t test; **** P < 0.0001; *** P < 0.001; ** P < 0.01; n.s – not significant.

Supplementary Figure S5 Enhancer-like region demarcated by eRNA loci drives activation of SNAI1 through cis-regulation. **(A)** RNA-seq analysis of CRISPR-activation using SCREEM2-tiling-locus(sgRNA10) in HBEC3-KT cells. Plotted is the ranked ordered gene set enrichment analysis of differentially expressed genes (p.adj <0.001), n = 3. **(B)** Heatmap showing selected EMT genes differentially expressed genes between NT and CRISPR-activation of SCREEM2-tiling-locus(sgRNA10) in HBEC3-KT cells. NT, non-targeting, n=3. **(C)** RNA-seq analysis of CRISPR-activation using SCREEM3-tiling -locus(sgRNA1) in HBEC3-KT cells. Plotted is the ranked ordered gene set enrichment analysis of differentially expressed genes (p.adj <0.001), n = 3 **(D)** Heatmap showing selected EMT genes differentially expressed genes between NT and CRISPR-activation of SCREEM3-tiling -locus(sgRNA1) in HBEC3-KT cells. NT, non-targeting, n=3.

Supplementary Figure S6 Genomic region proximal to SCREEM1 contains multiple lncRNAs. **(A)** UCSC genome browser view of the SCREEM1 locus and proximal genes (Top). Shown below are normalized read counts mapped to the locus from RNA-Seq data obtained from cells containing the following sgRNAs; SCREEM1 locus(sgRNA1) (green), SCREEM2-tiling-locus(sgRNA10) (blue), SCREEM3-tiling-locus(sgRNA1) (pink) Normalized CAGE counts mapped to the 5’ cap of SCREEM1, SCREEM2, and SCREEM3 are also shown for comparison (counts shown are from all samples in FANTOM5) (bottom). Note, RNA-Seq libraries for SCREEM1 sgRNA1 were made using polyA enriched RNA, whereas libraries for SREEM2 sgRNA10 and SCREEM3 sgRNA1 were made using total RNA (ribosomal RNA depleted).

Supplementary Figure S7 SCREEM1 transcript is dispensable for activation of SNAI1. **(A)** Transcript abundance of each SCREEM eRNA in different subcellular fractions of HBEC3-KT cells upon CRISPR-activation of SCREEM1 locus (sgRNA1). Plotted are relative values in each fraction from RT-PCR; NT, non-targeting. All values are mean ± SD with n = 3. Stats, unpaired t test; ** P < 0.01; **(B)** Expression of indicated eRNAs and SNAI1 following knockdown of respective eRNAS with shRNAs in HBEC3-KT cells expressing SCREEM1 (via CRISPR-activation) locus. Plotted are log2 fold change of delta-delta Ct values relative to scramble and EMC7 (housekeeping gene) from RT-PCR; scr, scramble. All values are mean ± SD with n = 3. Statistics, unpaired t test; ** P < 0.01; n.s – not significant. **(C)** Expression of indicated SCREEM eRNAs and SNAI1 following lentiviral over expression of respective eRNAS in HBEC3-KT cells. For comparison HBEC3-KT cells overexpressing SCREEM1 locus through SAM-mediated activation are shown (sgRNA1). Plotted are delta-delta Ct values relative to NT and EMC7 (housekeeping gene) from RT-PCR; NT, Non-targeting. All values are mean ± SD with n = 2. Statistics, unpaired t test; *** P < 0.001; ** P < 0.01; * P < 0.05.

Supplementary Figure S8 Monocytes express SNAI1 enhancer eRNAs and SNAI1, **(A)** Normalized FANTOM5 CAGE counts mapped to the 5’ cap of SCREEM1, SCREEM2, SCREEM3, TRERNA1 are shown for different monocyte activation conditions. CAGE counts for SNAI1 are included in each plot for comparison. **(B)** A USCS genome browser view of ChIP-Seq data from the BLUEPRINT epigenome consortium showing H3K27ac deposition across the SNAI1 cis-regulatory region in monocytes and macrophages. The region of the predicted enhancer is indicated by a horizontal black bar. Note the lack of enhancer marks spanning the predicted super-enhancer region in macrophages.
