# Supplementary figures and images for: A genome–wide CRISPR activation screen identifies SCREEM a novel SNAI1 super-enhancer demarcated by eRNAs
Source: Front Mol Biosci. 2023 Feb 27;10:1110445. doi: 10.3389/fmolb.2023.1110445 (PMC10009272; doi:10.3389/fmolb.2023.1110445)

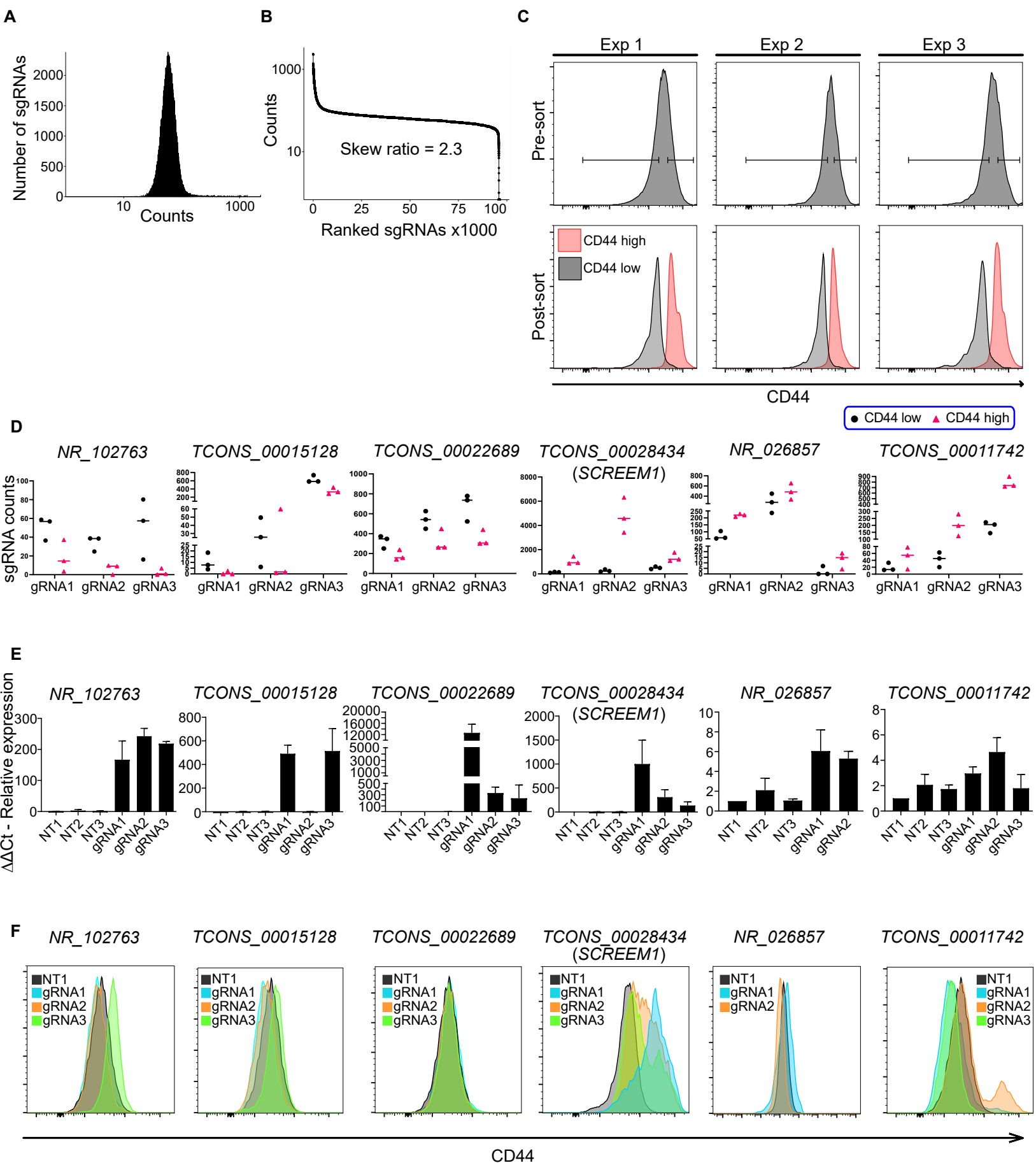

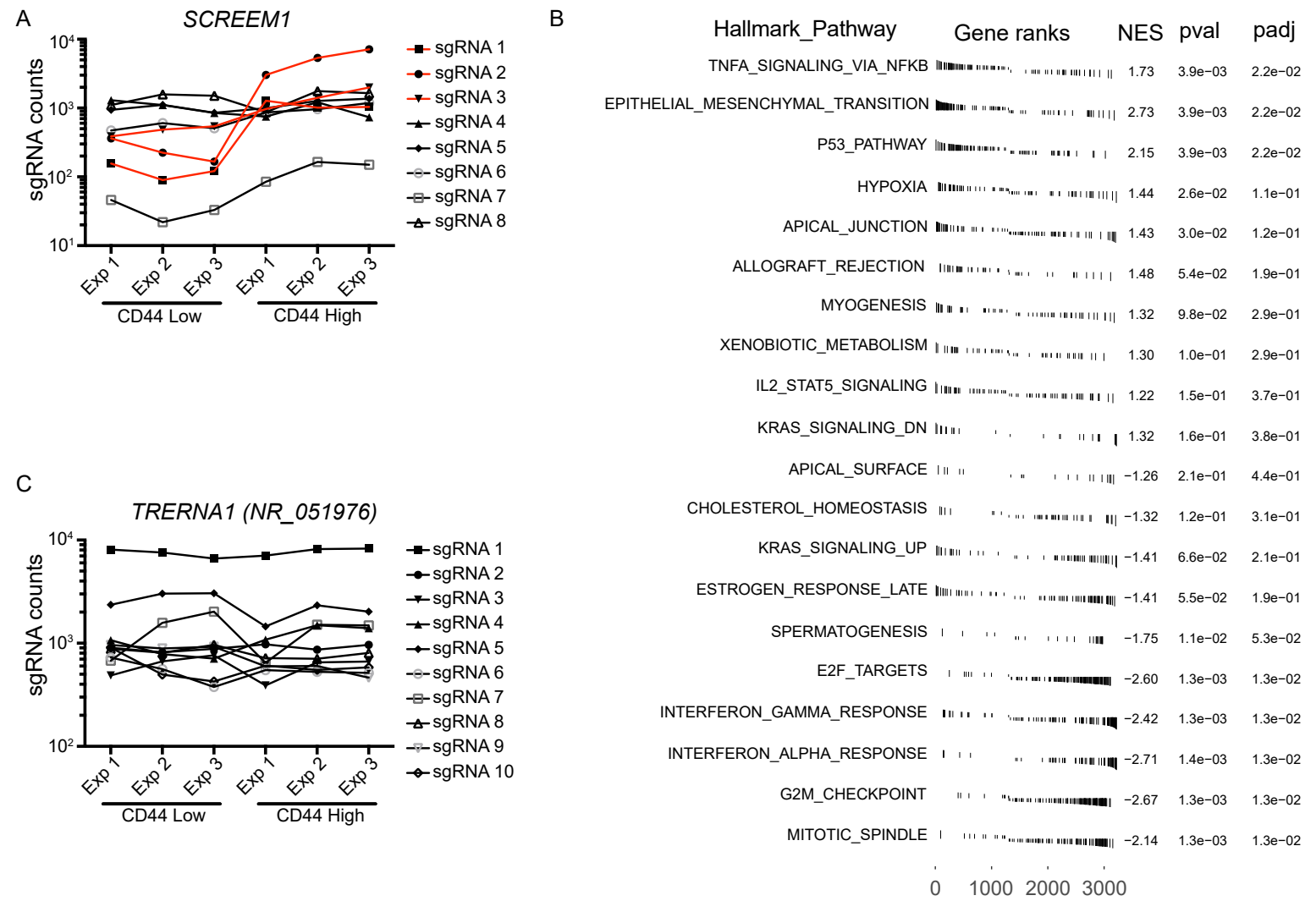

A

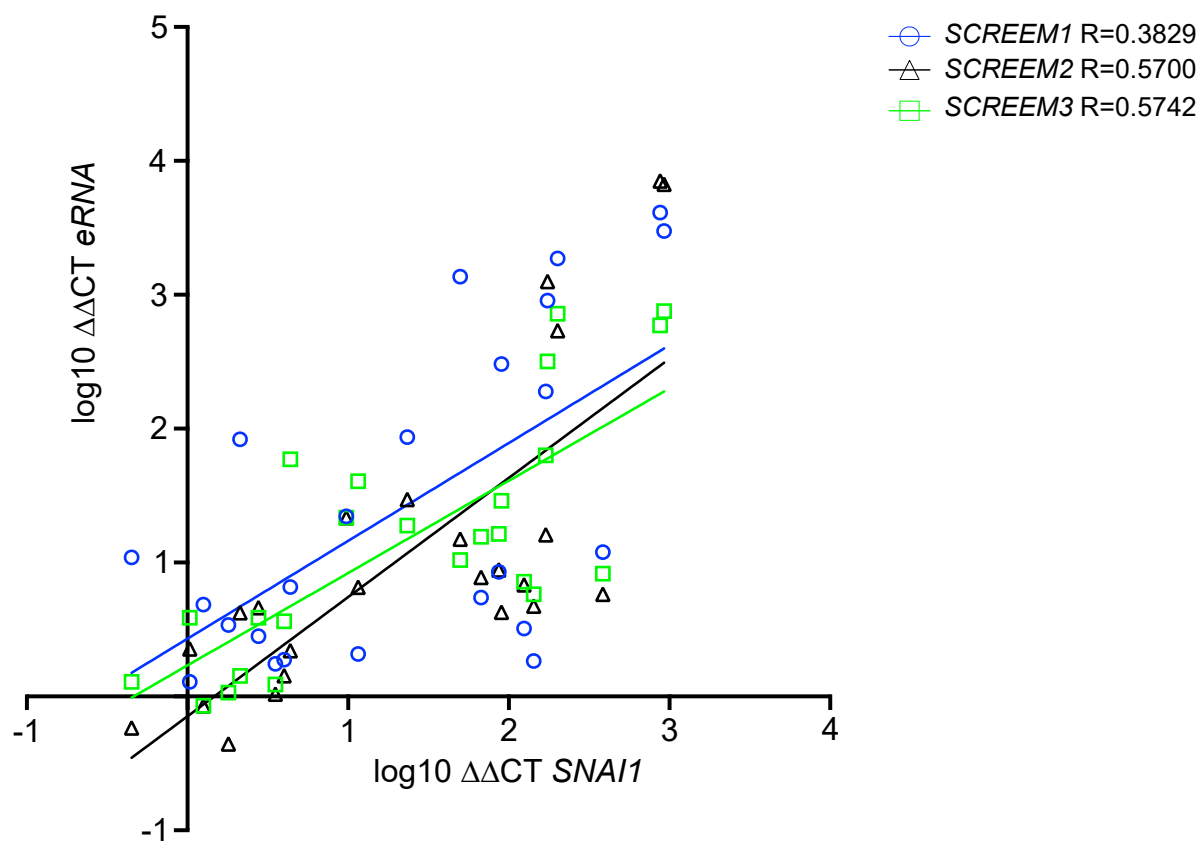

Uthaya Kumar et al Sup. Figure 3

A

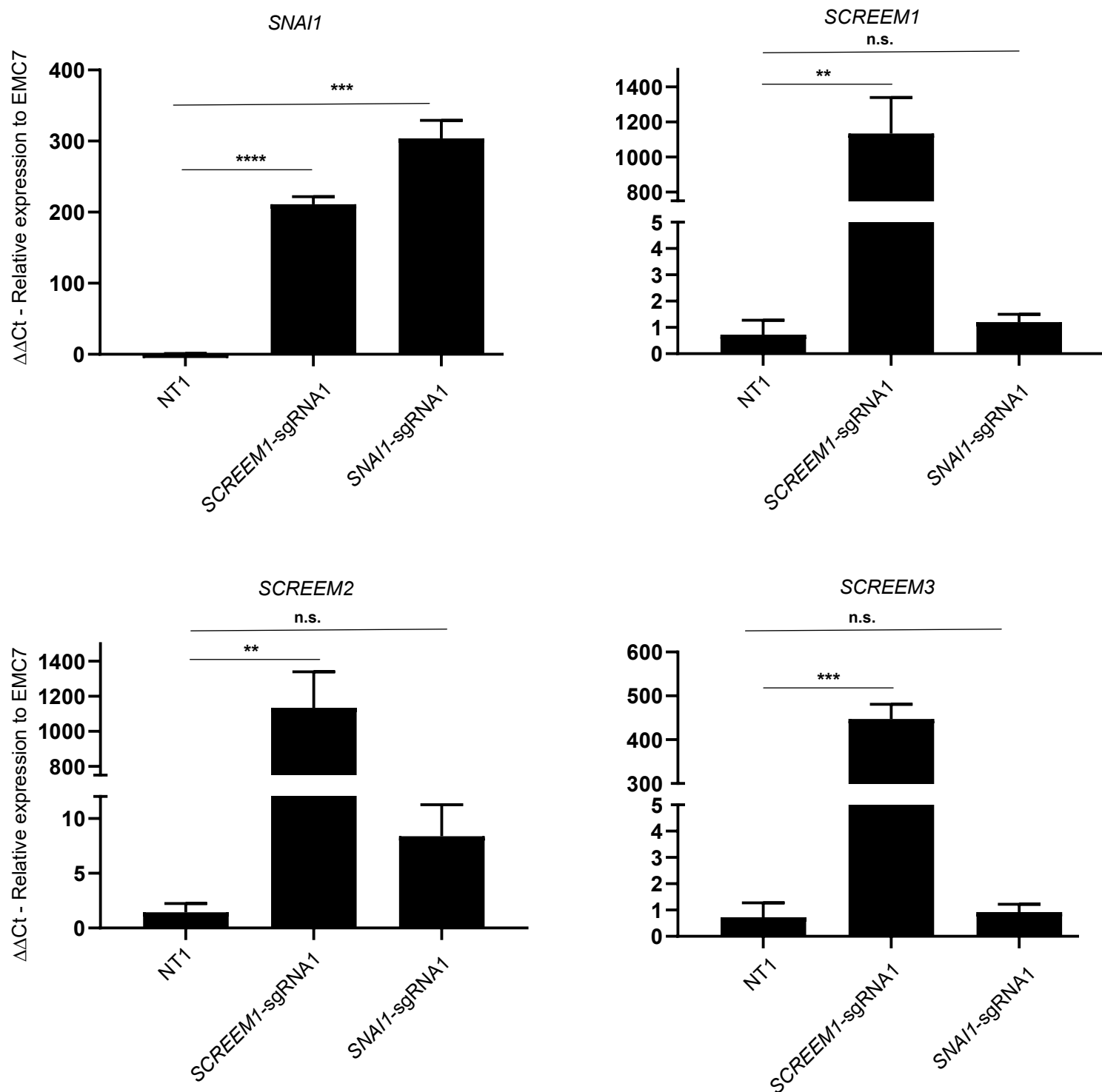

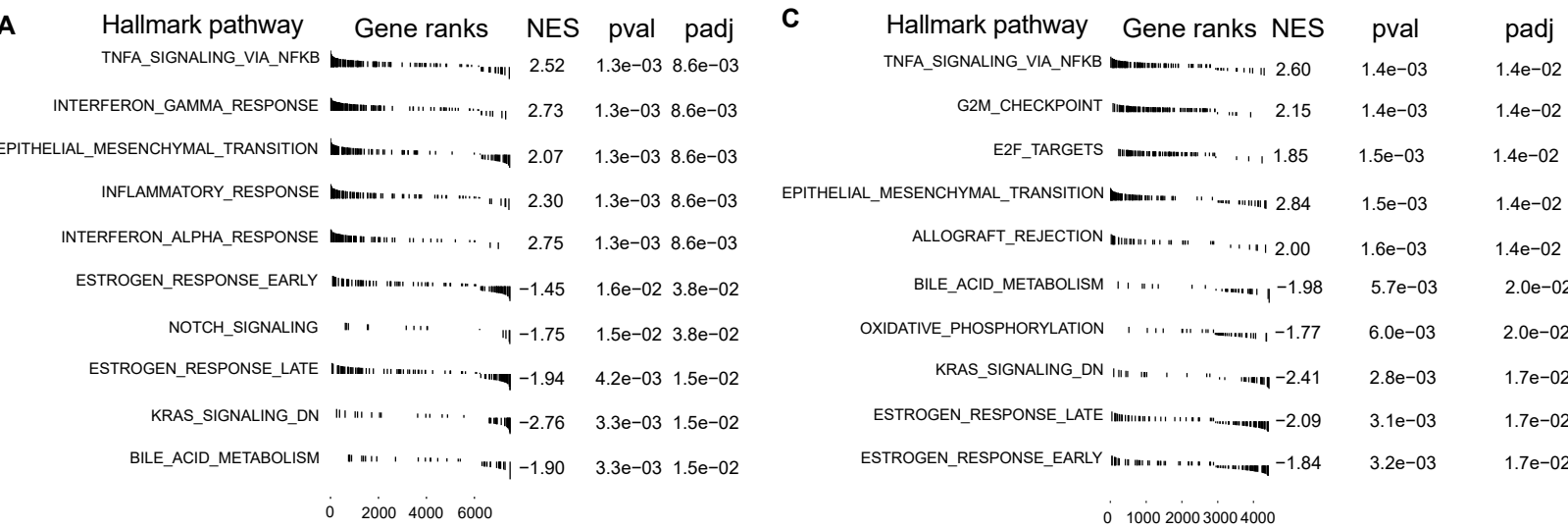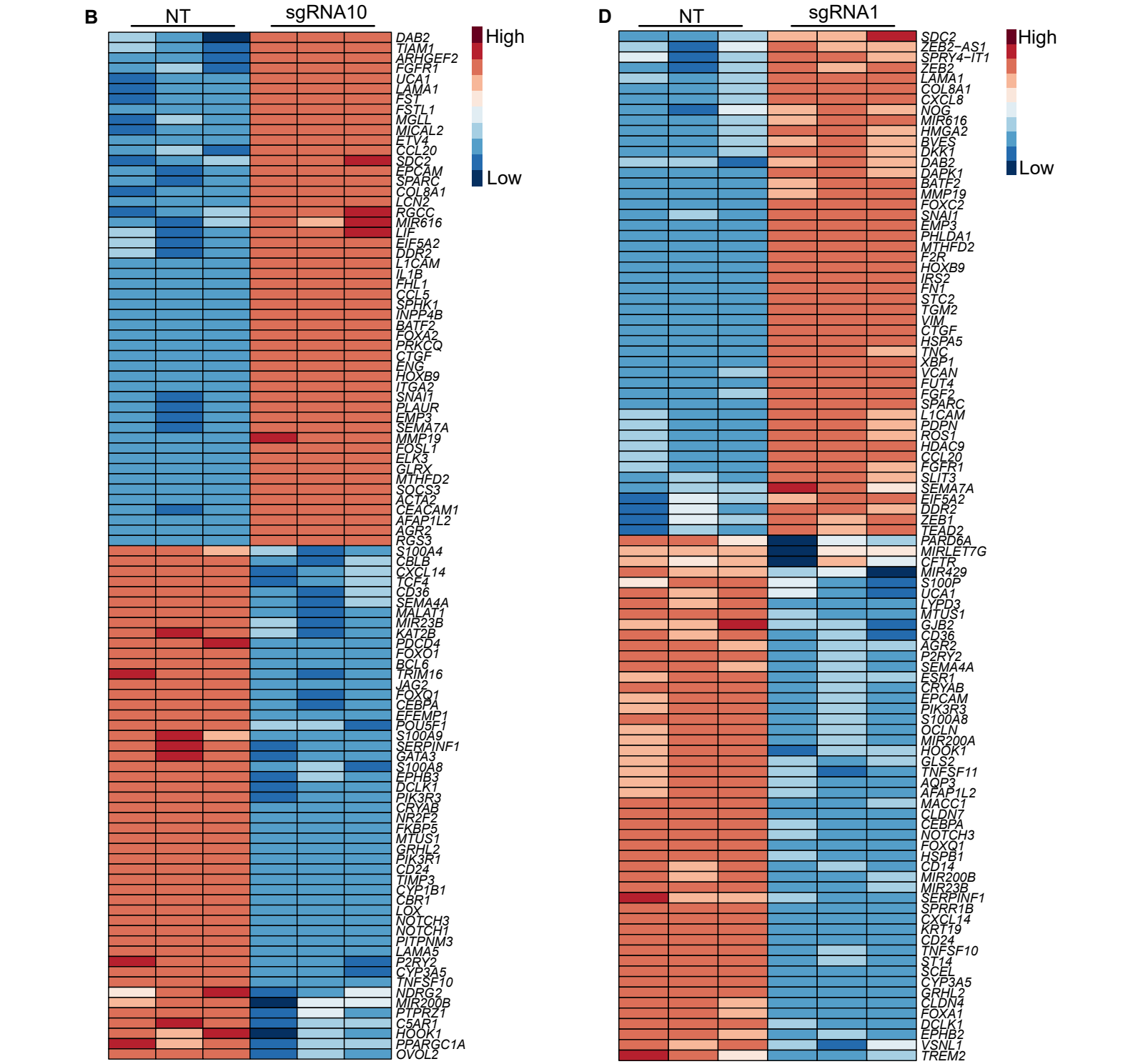

Uthaya Kumar et al Sup. Figure 5

A

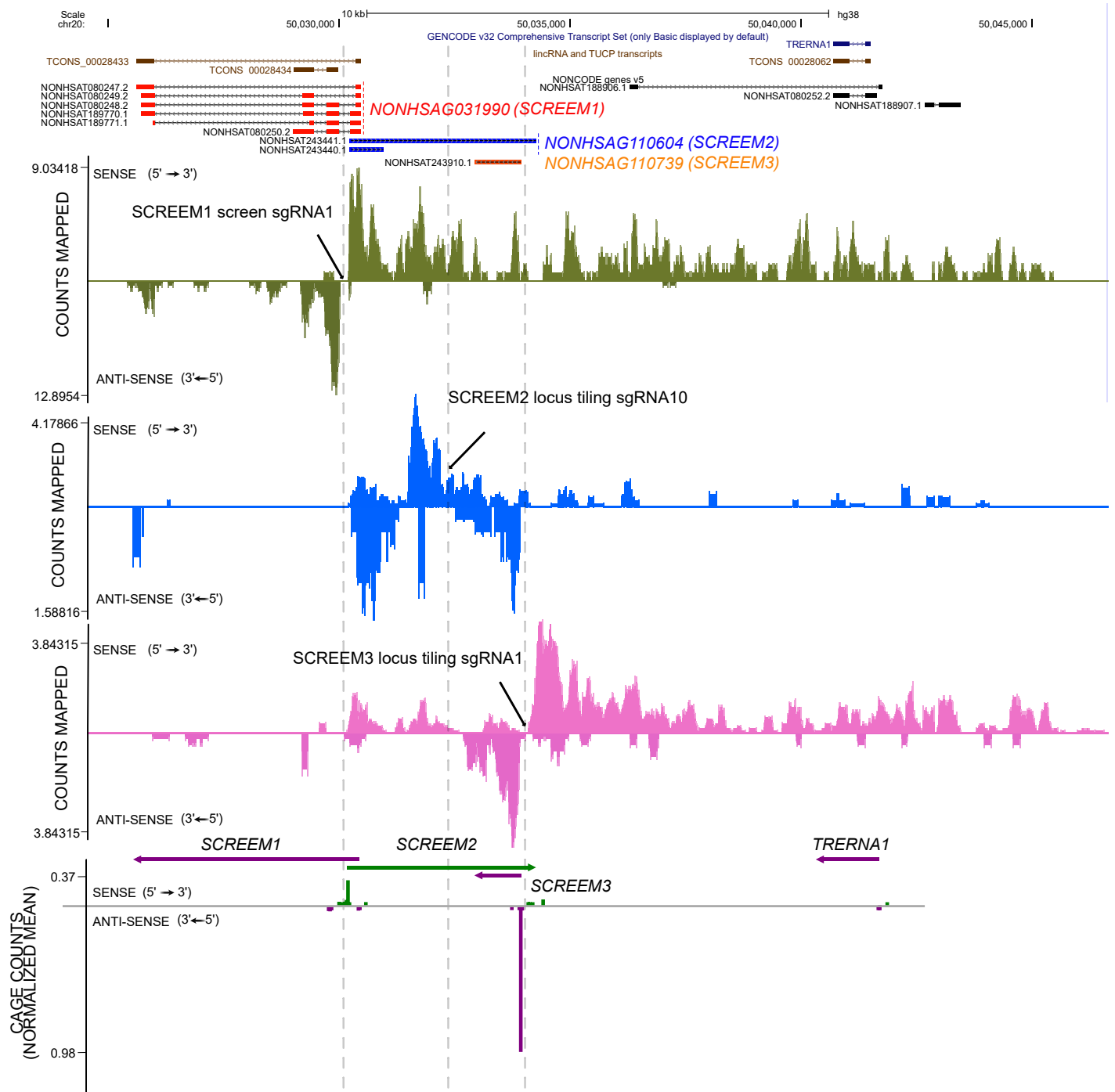

Uthaya Kumar et al Sup. Figure 6

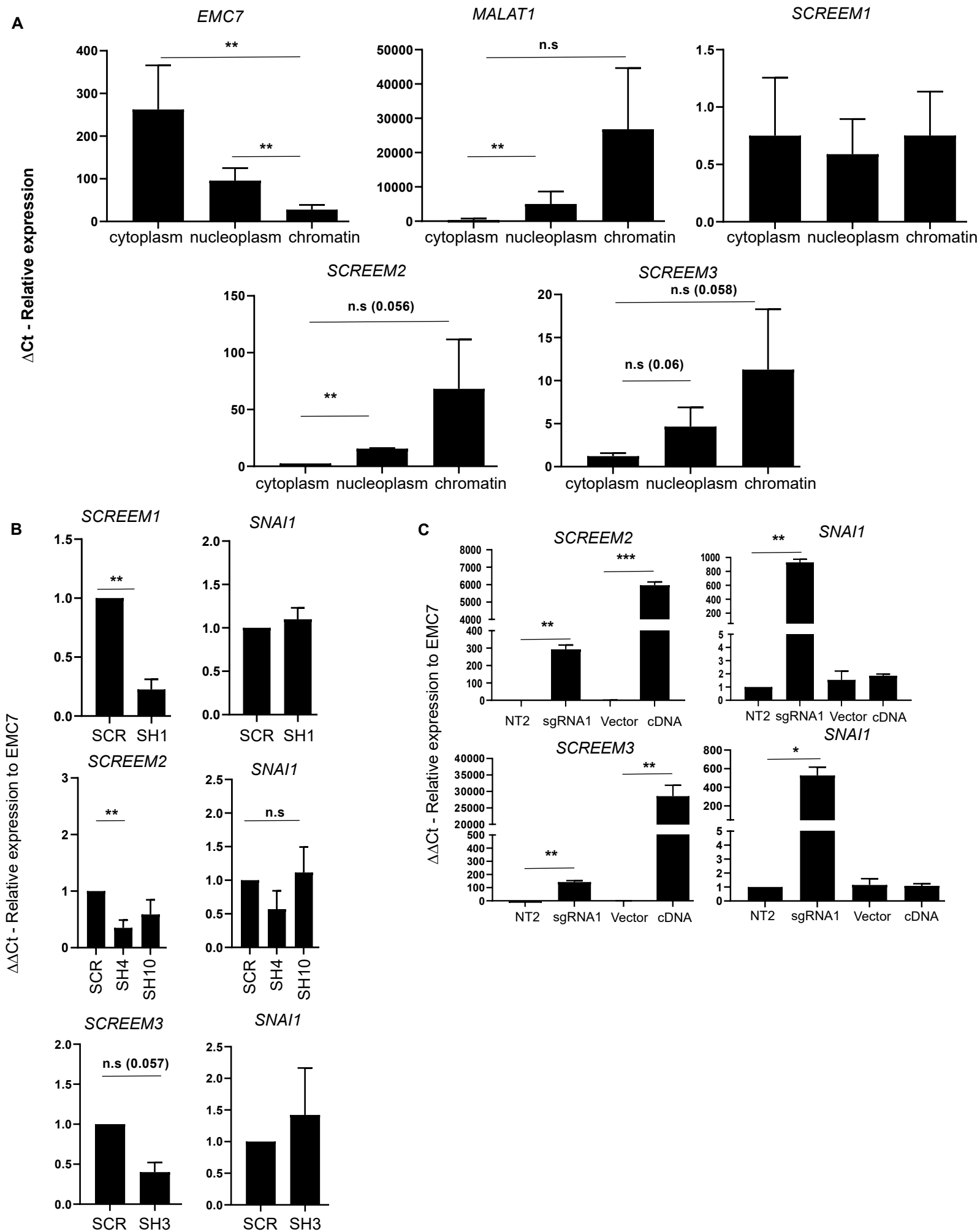

Supplement: Supplementary file 6 [file DataSheet1.PDF]
